# Supervised workplace learning in postgraduate training: a realist synthesis

**DOI:** 10.1111/medu.13655

**Published:** 2018-08-21

**Authors:** Anel Wiese, Caroline Kilty, Deirdre Bennett

**Affiliations:** ^1^ Medical Education Unit University College Cork Cork Ireland

## Abstract

**Context:**

This paper presents a realist synthesis of the literature that began with the objective of developing a theory of workplace learning specific to postgraduate medical education (PME). As the review progressed, we focused on informal learning between trainee and senior doctor or supervisor, asking what mechanisms occur between trainee and senior doctor that lead to the outcomes of PME, and what contexts shape the operation of these mechanisms and the outcomes they produce?

**Methods:**

We followed the procedures outlined in the RAMESES Publication Standards for Realist Synthesis. We searched the English‐language literature published between 1995 and 2017 for empirical papers related to informal workplace learning between supervisor and trainee, excluding formal interventions such as workplace‐based assessment. We made a pragmatic decision to exclude general practice training to keep the review within manageable limits.

**Results:**

We reviewed 5197 papers and selected 90. Synthesis revealed three workplace learning processes occurring between supervisors and trainees, each underpinned by a pair of mechanisms: supervised participation in practice (entrustment and support seeking); mutual observation of practice (monitoring and modelling), and dialogue during practice (meaning making and feedback). These mechanisms result in outcomes of PME, including safe participation in practice, learning skills, attitudes and behaviours and professional identity development. Contexts shaping the outcomes of these mechanisms were identified at individual, interpersonal, local and systems levels.

**Conclusions:**

Our realist theory of workplace learning between supervisors and trainees is informed by theory and empirical research. It highlights the two‐way nature of supervision, the importance of trainees’ agency in their own learning and the deleterious effect of fragmented working patterns on supervisor–trainee learning mechanisms. Further empirical research is required to test and refine this theory. In the meantime, it provides a useful framework for the design of supportive learning environments and for the preparation of supervisors and trainees for their roles in workplace learning.

## Abstract 2

The authors describe a realist theory of learning between supervisor and postgraduate trainee in the workplace, emphasising the role of individual, interpersonal, local and systems contexts in shaping key mechanisms and their outcomes.

## Introduction

The design of postgraduate medical education (PME) is underpinned by the premise that doctors learn through work.^1^, ^2^, ^3^, ^4^, ^5^ The clinical learning environment (CLE) provides the social, cultural and material context for PME.^6^ Supportive environments should afford trainees access to supervised participation in patient care, coaching, assessment and feedback, deliberate practice, teamwork, peer collaboration and observable models of practice.^7^, ^8^ Learners’ engagement with the affordances of the CLE leads to acquisition of practical knowledge, skills and attitudes, as well as to the development of professional identity.^9^, ^10^, ^11^, ^12^, ^13^, ^14^, ^15^, ^16^

Concerns have been expressed that changes in clinical environments might negatively impact workplace learning.^17^, ^18^, ^19^, ^20^ Underfunded,^21^, ^22^, ^23^ understaffed^24^ and overcrowded^25^, ^26^, ^27^ clinical environments present significant challenges for PME. The UK General Medical Council's Annual Training Survey Report 2016^28^ acknowledged the difficulties created by increased demand and shortage of staff. One in three supervisors reported not having sufficient time to fulfil their educational role. Self‐reported heavy trainee workloads were associated with a greater likelihood of trainees feeling forced to work beyond their competence and having patient safety concerns.^28^ Workplace bullying is more likely to occur when the workload is excessive^29^ and is frequently perpetrated by senior doctors.^30^ In addition, the effects of restrictions on duty hours, in place in North America and Europe, on learning, patient safety and workload remain uncertain.^31^, ^32^, ^33^, ^34^ To optimise conditions for learning, those tasked with the design and delivery of PME need to understand in detail the processes of medical workplace learning, and the influences of social and cultural contexts on those processes.^35^

Theories of workplace learning^35^, ^36^, ^37^, ^38^ and apprenticeship^39^ provide insights into PME in general terms; however, they do not provide detail about how PME works on the ground. This paper presents a realist synthesis that began with the objective of developing and refining a theory of workplace learning specific to PME derived from literature published on the topic. The project team is a multidisciplinary group of researchers, clinicians, health professions educators, experts in organisational behaviour, trainees and patient representatives. We chose a realist approach because it can capture both context and complexity, producing findings that are useful to policymakers and practitioners. The protocol for our realist synthesis^40^ initially posed the question: How, why and in what circumstances do doctors learn in clinical environments? Consistent with realist synthesis methodology, the focus of the review narrowed as it progressed through an iterative process of exploration of the literature and consultation with experts and stakeholders. We also considered findings emerging from our own concurrent research,^41^ which pointed to the central importance to learning of time spent working alongside senior doctors. Therefore, we chose to focus on the clinical and educational partnership between trainee and senior doctor or supervisor. Our refined review questions were as follows:

- What are the mechanisms occurring between trainee and supervisor or senior doctor that result in the outcomes of PME?
- What are the important contexts that shape the operation of these mechanisms and the outcomes they produce?

## Methods

### Conceptual orientation

Realist synthesis is an interpretative theory‐driven narrative summary of the literature describing how, why and in what circumstances complex social interventions work. Realist philosophy holds that outcomes of an intervention are context‐dependent. Realist synthesis translates the findings of empirical studies into context, mechanism and outcome (CMO) configurations, which state that in a certain context a particular mechanism generates a particular outcome.^42^ Using theory to identify CMO configurations focuses reviewers on the underlying and transferable aspects of programmes described.^42^ Theory is generated, tested and refined through this process.

### Procedures

This realist synthesis followed the steps and procedures outlined in the RAMESES Publication Standards for Realist Synthesis^43^ and associated training materials.^42^ Having defined the scope of the review as outlined above, we proceeded as follows.

#### Development of programme theory

Programme theory refers to assumptions about how an intervention is expected to work. Postgraduate medical education has a well‐developed programme theory, apparent in international standards and guidelines for its implementation; however, informal interactions between trainees and senior doctors during patient care are not well theorised in such documents. Drawing on these,^1^, ^2^, ^3^, ^4^, ^5^, ^44^ and on substantive theory,^35^, ^37^, ^38^, ^39^, ^45^, ^46^ we developed an initial programme theory for the informal learning that happens between supervisor and trainee in the course of patient care. Expressed in realist terms, PME is effective when:

- supervisors allow trainees to undertake increasingly complex patient care as their competence grows;
- supervisors monitor the work of trainees to match the complexity of work to the competence of the trainee and to provide trainees with feedback on their performance;
- trainees observe the practice of senior doctors and integrate what they see into their own practice, and
- trainees and supervisors discuss patient care.

We postulated that contexts at individual, interpersonal, local and systems levels would modulate the effectiveness of PME.

#### Search strategy

The complete search strategy is available in Appendix S1. In November 2015, we conducted broad searches employing both MeSH and free‐text terms relating to PME for English‐language literature published between 1995 and 2015 in the online databases Academic Search Complete, Australian Education Index, British Education Index, CINAHL, ERIC, PsycInfo, MEDLINE and SocIndex. As we focused the review, we added further searches relating to emerging contexts, mechanisms and outcomes, not adequately covered by the original searches, including ‘supervision’, ‘role‐modelling’ and ‘career choice’. We also hand‐searched key journals (*Academic Medicine, Advances in Health Sciences Education: Theory and Practice, Journal of Graduate Medical Education, Medical Education, Medical Teacher and Postgraduate Medical Journal*) for papers published between the years 2005 and 2015. Ongoing discussions with stakeholders and funders, with the aim of maximising the relevance of the findings, identified bullying as a focus for further searches. Additional studies were identified by searching for further publications by the authors of included papers. Finally, the bibliographies of all included papers, and relevant review papers, were screened. Searches were updated in August 2017.

#### Selection and appraisal of papers

Inclusion criteria were as follows:

1. papers related to informal learning between supervisor and trainee in PME in the clinical setting;
2. quantitative, qualitative and mixed‐method studies;
3. papers published in English, and
4. papers published between 1995 and 2017.

Exclusion criteria were as follows:

1. non‐empirical papers;
2. papers related to undergraduate medical education;
3. research on simulation or other non‐clinical interventions, and
4. papers relating to implementation or evaluation of formalised learning in clinical settings.

We excluded papers based on workplace‐based assessment (WBA) and on general practice (GP) training as we focused the review. Our focus on *informal* learning through work excluded WBA. Exclusion of GP was a pragmatic decision arising from the need to limit the volume of material to review and synthesise. Duplicates were removed and all papers were screened by title and abstract against these criteria. We reviewed full texts of the remaining papers for relevance to the focus of the review. A random sample (10%) was distributed to the wider research team to check concordance.

We considered whether papers were rich enough to contribute to the refinement of the programme theory and examined the credibility and trustworthiness of the data presented. In keeping with RAMESES guidelines, checklists, for example CASP, acted as sensitising influences but did not determine exclusion. Less rigorous studies may include trustworthy elements, which can contribute to CMO configurations. In this synthesis, such data were included only if they were supported by the findings of multiple other studies.

#### Data extraction

A data extraction sheet was designed and trialled. We recorded the title, authors, journal, country of origin, methodology and principal findings of each study. The authors read each included paper separately and together. We individually entered CMO configurations for each paper into an Excel sheet. These formed the basis for group discussion and the synthesis process.

#### Data analysis and synthesis

We categorised the papers according to their areas of focus and agreed CMO configurations within each paper. We then compared CMOs between papers, applying conceptual tools, juxtaposing, reconciling, adjudicating, consolidating and situating our findings.^42^ We were guided by our initial programme theory and by substantive theory.^35^, ^37^, ^38^, ^39^, ^45^, ^46^, ^47^ We sought predictable patterns (demi‐regularities) to determine how similar mechanisms act in different contexts to generate outcomes. Emerging findings were challenged, and contrary examples were sought in the data and in theory. This process allowed for contradictory outcomes to occur in particular contexts and for judgements of the strengths and weaknesses of research methods to be integrated into the synthesis, such that contradictory findings based on less rigorous research were disregarded. Discrepancies were discussed and resolved amongst the core research team with reference to the wider research team if necessary. AW, CK and DB each wrote thick descriptions of the agreed mechanisms, their associated outcomes and the influence of context on these, which were merged to form a final report. Throughout this process, the core team supported each other's reflexivity and maintained reflexive diaries. The multiprofessional nature of the wider team, including stakeholders and experts, also contributed to the reflexive process.

## Results

**Figure 1:**
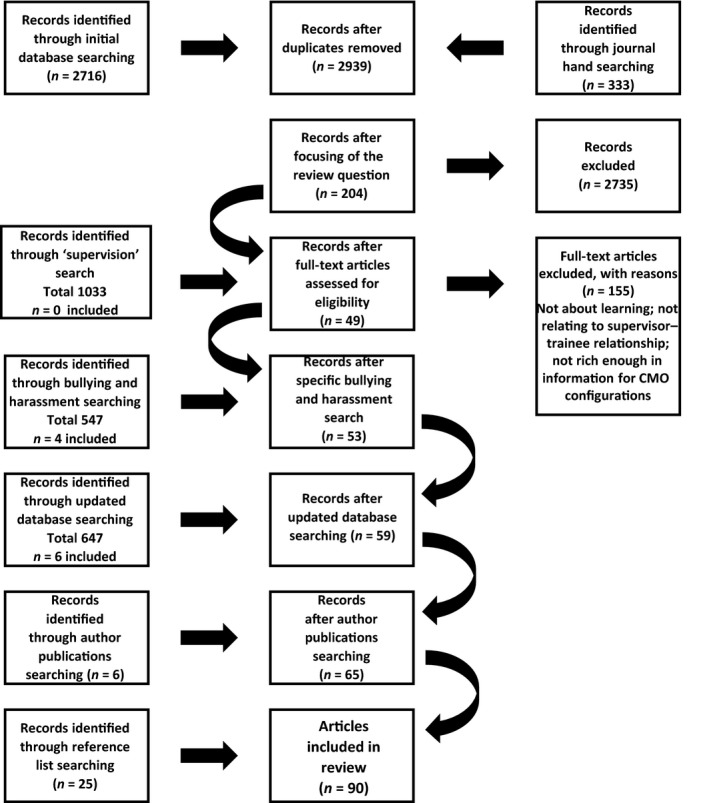
PRISMA (*p*referred *r*eporting *i*tems for *s*ystematic reviews and *m*eta‐*a*nalyses) flow diagram showing the literature retrieval process. CMO = context, mechanism and outcome

The PRISMA flow chart in Fig. 1 illustrates the process that led to the selection of 90 papers for inclusion in the review (Appendix S2). Sixty‐seven were qualitative studies, 19 were quantitative studies and the remainder used mixed methods. The USA, Canada, the UK and the Netherlands accounted for 80 of the 90 included studies. Almost half (38/90) of the papers reported on multiple specialties and the remainder covered a range of individual specialties. A small minority of included studies (*n* = 4) were judged as being of lesser quality, and their findings were used only to confirm those found in more rigorous studies.

**Figure 2:**
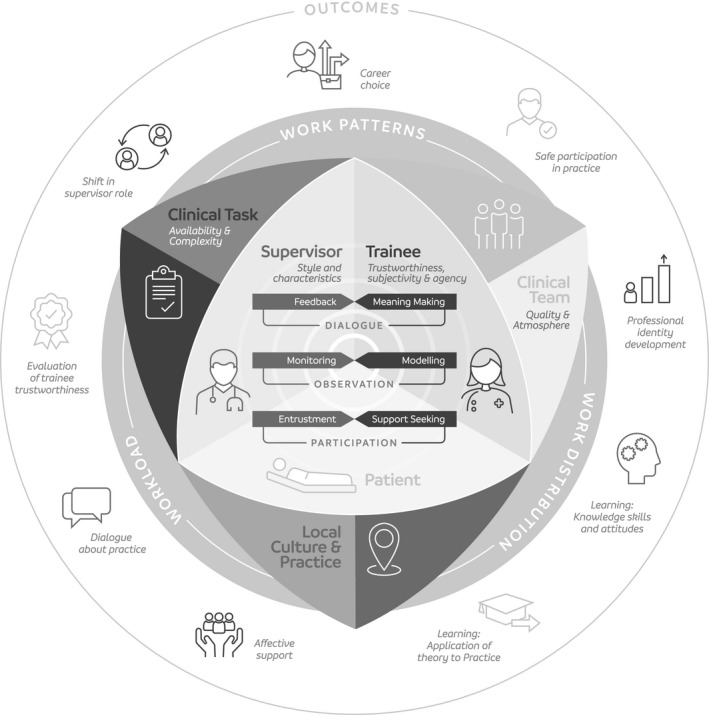
A realist theory of supervised workplace learning

Our synthesis of these studies is summarised in Fig. 2.

Postgraduate medical education happens in triadic relationships among supervisors, patients and trainees as represented by the triangle at the centre of the schematic. We have focused on the supervisor–trainee dimension. We identified three interrelated processes occurring informally between supervisors and trainees in the course of patient care. These were *supervised participation in practice, mutual observation of practice* and *dialogue about practice*. A pair of mechanisms underpins each of these processes (Table 1). Individual and interpersonal contexts that influence these mechanisms are shown in the innermost triangle. Contexts relating to the local clinical environment are shown at the next level. These are embedded in a further layer of health systems contexts. The way in which mechanisms operate and the outcomes they produce are shaped by these multilayered contexts. The outcomes of PME are shown in the outermost layer. These and other outcomes act in turn as both contexts and mechanisms in themselves, thus creating a cycle in which positive outcomes enhance the outcomes of future interactions. The total number of papers that contributed to the description of CMO configurations for each mechanism is shown in Table 1.

**Table 1:** Supervisor–trainee processes in postgraduate medical education, their underpinning mechanisms and papers contributing to individual, interpersonal and local contexts, mechanisms and outcomes (CMOs) for each

Process	Mechanisms	Papers, contributing *n*
Supervised participation in practice	Entrustment	31
	Support seeking	17
Mutual observation of practice	Monitoring	14
	Modelling	33
Dialogue during practice	Meaning making	33
	Feedback	21

We now describe each mechanism and its outcomes and then outline in detail how individual, interpersonal and local contexts shape their operation and outcomes. We then address the overarching health systems contexts that impact all supervisor–trainee mechanisms. In the following sections, mechanisms and their major outcomes are shown in bold. Contexts, mechanisms and outcomes are indicated in the text by (C), (M) and (O), as appropriate.

### Supervised participation in practice

The degree to which trainees participate in practice, and the level of supervision applied, hinge on the mutually interdependent mechanisms of *entrustment* (M) and *support seeking* (M). The interplay between these mechanisms has been likened to a dance, with both supervisor and trainee leading in turns.^48^ There is extensive literature on *entrustment*, but much less on *support seeking*.

#### Entrustment: mechanism and outcomes

Programme theory suggests that PME is effective when trainees are trusted with increasingly complex tasks as their competence grows. Although the term *entrustment* (M) is often associated with formal assessment,^49^, ^50^ we refer here to informal ‘point of care’ evaluation,^51^, ^52^ leading to ad hoc decisions regarding what a trainee can be relied upon to undertake safely.^53^ *Entrustment* (M) gives trainees the opportunity for *safe participation in practice* (O), allowing them to manage emerging problems, supported by a safety net of supervision.^8^, ^54^, ^55^, ^56^ Thus, participation (O) can be viewed as an outcome of *entrustment* (M) and as a mechanism in itself, which leads to growth in knowledge, professional identity development^8^, ^57^, ^58^, ^59^, ^60^, ^61^, ^62^ and engagement in progressively more complex work.^48^, ^61^, ^63^ Participation (O) and its precursor *entrustment* (M) prepare trainees for future independent practice.^52^, ^63^, ^64^, ^65^ *Entrustment* (M) also leads to a *shift in supervisor role* (O) as oversight fades, moving away from direct patient care towards acting as a consultant to the trainee.^51^, ^53^ The supervisor is more relaxed (O), which enhances team relationships (O/C),^48^, ^53^, ^63^ in turn fostering greater *entrustment* (M).^53^, ^66^, ^67^ These positive outcomes are balanced against supervisor vulnerability (O) arising from the risk of the trainee being involved in an adverse event (O).^52^, ^56^

#### Individual and interpersonal contexts for entrustment: supervisory style, trainee trustworthiness and trainee agency

Supervisors must balance trainees’ autonomy in practice (O) against the provision of safe care (O).^48^, ^56^, ^63^, ^67^ Supervisory styles vary from that of the micromanager (C), who entrusts very little clinical responsibility to trainees (O), to that of the minimalist supervisor (C), who allows trainees almost total autonomy (O).^53^, ^55^, ^68^, ^69^ A micromanaging supervisor (C) leads to reduced *participation in practice* (O),^68^, ^70^ creating frustration, lack of motivation, feelings of being undervalued^55^, ^71^ and inadequate preparation for independent practice.^52^, ^62^ The minimalist approach (C) leads to excessive trainee responsibility and unsafe *participation in practice* (O), leaving trainees feeling overwhelmed, fearful, confused about their role and concerned that their limitations might not be recognised (O).^55^, ^62^, ^72^, ^73^, ^74^ Intermediate approaches to supervision (C) can support progressive independence (O) and adapt to context.^69^ Trainees prefer a style that accounts for trainee needs and stage of training.^55^

When a supervisor works with a new trainee (C), ‘presumptive’ *entrustment* (M)^52^, ^53^ is based on the trainee's stage of training (C) and reports of his or her competence (C).^66^, ^67^ Supervisors also seek their own evidence of trustworthiness (C)^53^, ^56^, ^66^, ^71^ so that over time *entrustment* (M) becomes grounded in first‐hand knowledge of the trainee's abilities observed during clinical work (C).^53^, ^56^, ^63^, ^66^, ^67^, ^75^ In procedure‐based specialties (C), *entrustment* (M) is more explicit than in other types of practice. In this context, multiple observations (C), experience, in terms of numbers of procedures previously undertaken (C), and the trainee's own ‘comfort’ (C)^60^, ^62^, ^66^, ^76^ trigger *entrustment* (M).

Similarly, evidence of professional skills and behaviours (C) enhances *entrustment* (M). These include leadership and management skills (C),^53^, ^66^, ^67^ accountability (C),^56^ conscientiousness (C),^51^, ^67^ appropriate confidence,^66^, ^67^ awareness of one's own limits (C)^8^, ^51^, ^53^, ^56^, ^63^, ^66^, ^77^ and acceptance of criticism (C).^67^ Inhibitors of *entrustment* (M) include red flags about professionalism or over‐confidence (C).^53^, ^67^

Trainee agency (C), the degree to which trainees choose to take an active role in their own learning, is also an important context. Trainees who signal a desire to become more autonomous (C) trigger *entrustment* (M).^53^, ^56^, ^62^, ^63^, ^70^, ^71^ Knowing individual supervisors’ preferences, and working in accordance with them (C), also increases participation in practice (O).^78^ More passive trainees (C)^71^ and those actively requesting help (C) inhibit *entrustment* 63(M), leading to fewer opportunities and closer supervision(O).^66^

#### Local contexts for entrustment: clinical task and local culture and practice

Low‐stakes routine tasks (C) encourage *entrustment* (M).^48^, ^53^, ^60^, ^67^, ^71^, ^76^ Clinical complexity (C), family or ethical dilemmas (C), interdepartmental and interdisciplinary collaboration (C), urgency and severity of illness (C), and transitions of care (C) have been identified as contexts leading to greater supervisor involvement (O).^52^, ^53^, ^54^, ^62^, ^63^, ^66^, ^67^ Paradoxically, complexity (C) may also trigger *entrustment* (M)^53^, ^54^, ^63^ for senior trainees (C).

In smaller hospitals (C), trainees participate more (O), whereas in larger hospitals (C) they are more likely to be observers (O).^54^, ^79^ The location of the supervisor relative to the trainee is an important local context. Proximity (C) enhances *entrustment* (M) because the supervisor is nearby to step in if needed.^66^, ^67^ Paradoxically, at night, when supervisors are often off site (C), trainees are also more likely to be trusted (M) with clinical care activities (O).^62^, ^64^, ^66^, ^67^

#### Support seeking: mechanism and outcomes

Programme theory suggests that PME is effective when trainees seek support when they need it but work autonomously when they do not. *Support seeking* (M) refers to a trainee‐initiated approach to a senior doctor with the objective of gaining input to patient care. It acts as a counterbalance to *entrustment* (M). When a trainee seeks support (M), he or she may be trusted with the continuing management of the patient (O) or the supervisor may step in and take over care to a greater or lesser degree (O).^75^ Trainees’ *safe participation in practice* (O) is an outcome of effective support seeking. Failure to seek support results in compromised patient safety (O).^80^, ^81^, ^82^ *Support seeking* (M) also supports *professional identity development* (O), allowing trainees to advertise themselves as responsible professionals.^81^, ^83^

#### Individual and interpersonal contexts for support seeking: supervisory style, trainee subjectivity and trainee agency

The decision to seek support is complex and highly context‐dependent.^81^, ^83^, ^84^ Supervisor approachability and availability (C) are key to providing a safe environment,^59^, ^67^, ^70^, ^81^ which, along with clear communication regarding when to call (C), encourages *support seeking* (M).^85^ Intolerance, reference to being otherwise occupied (C) or speaking negatively about having been called on previous occasions (C), inhibits *support seeking* (M);^83^, ^85^ this may apply only when the decision to seek support is not clear cut (C).^84^

Trainee subjectivity, how trainees interpret and understand situations, influences *support seeking* (M). Thus, their perceptions (C) of the likely outcome of *support seeking* (M) may trigger or inhibit the mechanism. Trainees worry (C) about loss of trust, autonomy or reputation,^70^, ^82^ negative evaluations^81^, ^83^, ^86^ and negative comparisons with peers^74^ if they seek support (M) inappropriately. They may be reluctant to contact seniors (M) at night (C) as this might antagonise the latter or make them less effective the following day.^81^, ^84^ Trainees want to enact the identity of an independent doctor (C) and this also shapes decisions to seek support.^61^, ^74^, ^81^, ^83^, ^84^ Although support seeking (M) allows trainees to demonstrate appropriate professionalism (O), not seeking support (M) may also support professional identity (O).^83^

Trainees can influence the outcome of support seeking through the use of rhetorical strategies (C), which support credibility and facilitate trust to continue care (O).^83^ Inaccuracies in the language used (C) or discrepancies in the clinical information provided (C) may inhibit *entrustment* (M).^75^ When triggered too frequently (C), *support seeking* (M) can negatively impact supervisor evaluations and threaten credibility (O).^59^, ^83^, ^84^

#### Local contexts for support seeking: clinical task, team and local culture and practice

*Support seeking* (M) is more likely when there are serious consequences for the patient (C),^83^, ^84^ or when the problem falls outside the scope of practice of the trainee.^74^, ^83^ Scope of practice for a particular level of trainee varies across departments and sites (C).^54^, ^64^, ^66^ Nonetheless, some activities (C) are reported as always requiring supervisor input (O).^67^, ^68^, ^83^ Explicit declaration of availability within the team to help the trainee (C) enhances *support seeking* (M).^67^ However, even in the presence of a supportive team (C), some trainees still find it difficult to ask busy colleagues (C) for support (M).^80^, ^81^

### Mutual observation of practice

Supervisors and trainees observe one an other's practice as they work alongside each other. This process of mutual observation is underpinned by the *monitoring* and *modelling* mechanisms.

#### Monitoring: mechanism and outcomes

Programme theory suggests that PME is effective when supervisors monitor the work of trainees to match the complexity of work to the competence of the trainee and to provide trainees with feedback on their performance. Trainees’ work is continually monitored (M) by their supervisors, with the primary objective of ensuring safe patient care (O).^51^, ^61^, ^75^ Informal observation of trainees’ practice (M) for primarily educational reasons is uncommon.^64^, ^87^, ^88^ When routine monitoring (M) raises concerns (C), increased supervisory vigilance results (O) and the supervisor may step in to ensure patient safety (O).^75^ Effective *monitoring* (M) therefore leads to trainees’ *safe participation in practice* (O).^75^ Routine oversight includes direct observation of clinical activities,^53^, ^56^ focusing on what is valued (e.g. technical skills in surgery).^56^, ^87^ More often trainee work is indirectly monitored. Supervisors monitor work that is not visible to them through dialogue with trainees about patient care.^61^, ^75^ ‘Backstage oversight’ involves double‐checking clinical findings and reading patient records.^51^, ^61^, ^67^ *Monitoring* (M) in this way leads to *evaluation of trainee trustworthiness* (O), which is an important context for *entrustment* (M). *Monitoring* (M) may also lead to *dialogue about practice* (O) between supervisor and trainee. Dialogue, described later in this paper, has its own important educational outcomes.^75^, ^87^

#### Individual and interpersonal contexts for monitoring: supervisory style and trainee subjectivity

Supervisors apply varying standards (C) when *monitoring* (M) trainees’ work, which shape their *evaluation of trainee trustworthiness* (O).^53^, ^78^, ^89^ Trainees recognise this and adjust their performance to match individual supervisor requirements (O).^90^ For trainees, being directly observed (M) conflicts with the desire to be efficient and autonomous (C).^87^ The presence of an observer (C) can alter performance and leave the trainee feeling undermined in his or her relationship with the patient (O).^87^, ^90^ If a supervisor takes over (O), the trainee may lose confidence and feel undercut (O).^48^, ^63^ Greater frequency of direct observation (C) may lead to greater comfort (O).^90^

#### Local contexts for monitoring: clinical task and clinical team

A sick patient, change in condition or a need for critical decision making (C) may lead to increased supervisory vigilance (M), regardless of the trainee's competence (C).^75^ Other members of the clinical team, such as nursing staff, may contribute ‘backstage’ information (C) to support *monitoring* (M).^75^ However, Hauer et al.^53^ found that input of this nature was infrequently mentioned as a source of information for *entrustment* (M).

#### Modelling: mechanism and outcomes

Programme theory assumes that PME is effective when trainees observe the practice of senior doctors and integrate it into their own. We define *modelling* (M) as observing the practice of any senior doctor and integrating that into one's own practice (O).

Trainees are exposed to an array of examples of how to speak, act and think like a doctor.^89^, ^91^, ^92^, ^93^ They observe senior doctors communicating with patients and families, undertaking physical examinations, making clinical decisions and dealing with professionalism issues.^88^, ^91^, ^92^, ^94^, ^95^ The practice of any more senior doctor observed by the trainee may be modelled.^48^, ^88^, ^96^, ^97^ Modelling may be made explicit by the *meaning making* (M) mechanism, described in the next section, when senior doctors think aloud and reflect with learners on modelled behaviours.^58^, ^65^, ^89^, ^92^, ^96^, ^98^ However, typically modelling occurs without dialogue, strategy or even consciousness on the part of the model.^58^, ^96^, ^98^, ^99^ *Modelling* (M) involves watching, seeing and hearing and may also include reflection and contemplation of future practice.^48,58,64,77,95,99–101^ It may be a deliberate learning strategy,^92^ but it usually occurs unconsciously.^99^

*Modelling* (M) leads to *learning, knowledge, skills and attitudes* (O) across a range of competences.^57^, ^92^, ^95^ It is particularly associated with learning in the informal curriculum,^91^, ^99^, ^102^ in domains such as communication,^88^, ^92^, ^95^, ^96^, ^102^, ^103^ collaboration,^88^, ^93^, ^96^ professionalism,^58^, ^88^, ^93^, ^96^, ^99^, ^104^ humanism^98^ and leadership (O).^93^, ^105^ *Modelling* (M) also leads to *learning, application of theory to practice* (O),^92^, ^96^ by allowing trainees to observe variations in practice amongst senior doctors (C).^89^, ^91^ Trainees develop their own ‘style’ of doctoring (O) by observing (M) these multiple ways of ‘doing’ (C) and adapting what they see for their own use.^88^, ^89^, ^92^, ^95^, ^96^, ^106^, ^107^

Senior doctors also model career possibilities for trainees. Their satisfaction with their job, stress levels and work–life balance (C) may lead to trainees being attracted to or deterred from specific specialties (O).^108^ Encountering role models, positive and negative, within specialties (C) has been identified^109^, ^110^, ^111^ as one factor that influences *career choice* (O).

#### Individual and interpersonal contexts for modelling: supervisor characteristics and trainee subjectivity

Several papers identify features of positive models (C), individuals who are highly professional and encourage similar behaviour (O),^58^ thus triggering *modelling* (M). Characteristics identified include clinical excellence with an emphasis on the psychosocial aspects of care,^97^, ^102^, ^106^, ^112^ strong interpersonal skills, leadership, integrity, teaching skills,^97^, ^106^, ^113^, ^114^ an enthusiasm for work^91^, ^97^, ^106^ and awareness of one's own strengths and limitations^96^, ^98^ (C). By contrast, senior doctors who are quiet, impatient, over‐opinionated (C) or lacking in collaborative and humanistic attitudes (C) have been identified as negative models (O).^97^, ^112^ Trainees avoid interactions (O) with negative models (C).^92^, ^95^ Other literature suggests that trainees’ subjectivities (C) lead them to select models (M) with whom they share values, attitudes and styles of communication.^57^, ^92^ Perceptions of a good model change (C) as the trainee becomes more senior (C),^101^, ^106^ with a shift in focus from modelling knowledge and skills to communication and empathy (O).^95^

Negative role modelling (M) may occur when trainees observe unprofessional behaviour or negative traits such as cynicism in supervisors (C).^91^, ^115^ Rejection of negative behaviour and the active exclusion of it from one's own repertoire (O) is one outcome of such an observation;^58^, ^91^, ^92^, ^95^ however, failing to recognise it as unprofessional and adopting it (O) is another.^58^

### Dialogue about practice

In the course of daily work, supervisors and trainees talk about practice, in terms of both the content of clinical work and trainees’ performance in practice. This dialogic process is underpinned by the mechanisms, *meaning making* and *feedback*. Unlike the mechanisms discussed under Supervised participation in practice and Mutual observation of practice, *meaning making* and *feedback* can be either supervisor‐ or trainee‐led. Both are sense‐making activities, but each has a distinct focus.

#### Meaning making: mechanism and outcomes

Programme theory suggests that PME is effective when supervisors and trainees make sense of clinical work together through dialogue. *Meaning making* (M) is typically centred around shared clinical reasoning through iterative questioning and answering, which aims to stimulate critical thinking and to draw out underlying presumptions.^48,64,92,94,95,116–119^ Trainees present a case in a structured format.^61^, ^83^, ^94^, ^116^ Supervisors use questions to orient trainees to salient information, to probe background knowledge and to clarify the management to date.^64^, ^94^, ^117^, ^118^ Pauses allow trainees to reconsider incorrect responses.^120^ Gaps or inconsistencies in the case presentation trigger knowledge‐related probing questions, sometimes leading to a specific teaching point.^54^, ^117^ Trainees ask questions to fill specific knowledge gaps and to explore supervisors’ experiences with similar cases.^54^, ^118^ They may express uncertainty about the meaning of findings or outline a tentative management plan,^66^, ^118^ reasoning aloud about complex aspects of a case.^54^, ^95^, ^118^ Non‐verbal communication is also important, with gestures, mannerisms and intonations being used to express uncertainty.^118^, ^121^ Supervisors offer unsolicited knowledge^54^, ^118^, ^121^ using linguistic devices such as metaphor, simile and storytelling.^118^, ^121^ Supervisors interpret theoretical knowledge in the context of the clinical situation for trainees.^65^, ^118^

*Meaning making* (M) with trainees leads to *evaluation of trustworthiness* (O). Language cues, structure and delivery of case presentations are proxy indicators (C) of clinical competence.^51^, ^116^ Presentation of a comprehensive plan (C) allows the supervisor to estimate the trainee's insight into possible complications (O).^51^, ^66^, ^117^

Through *meaning making* (M) trainees learn how to think about clinical problems, moving beyond guidelines and protocols to *learning, application of theory to practice* (O).^64^, ^92^ Interpretation, construction of meaning and reflection with the supervisor allow the integration of new experiences (O).^57^, ^77^ *Meaning making* (M) stimulates reflection, sharpens awareness and leads to increased concentration in future practice situations (O).^54^, ^64^, ^68^

*Meaning making* (M) leads to *professional identity development* (O). Trainees enact the values of medicine, such as logical reasoning based on scientific principle, advertising their professional identity (O) by articulating technical medical language fluently.^61^, ^116^ Trainee self‐confidence is enhanced (O) through *meaning making* (M) when trainees' approaches match those of supervisors (C).^54^, ^64^

There are also more informal exchanges, when supervisors discuss worries, errors or the burden of paperwork.^91^ Some deliberately exhibit vulnerability and uncertainty, demonstrating how they deal with these feelings.^91^ *Meaning making* (M) in this fashion can provide *affective support* (O) to trainees. Debriefing (M) after errors or negative outcomes is critical in helping trainees to cope (O),^122^ as are maintaining confidence, placing events in perspective and modelling reflective practice (O).^57^

#### Individual and interpersonal contexts for meaning making: supervisory style and trainee agency

Supervisors create a safe environment (C) for *meaning making* (M) by acknowledging that they don't know everything (C), and enhancing team rapport and exchange (O).^73^, ^91^ A collaborative focus on the learner's needs (C),^73^, ^123^ particularly for senior trainees (C),^73^, ^121^ leads to effective *meaning making* (M). Failure to involve trainees in discussion and decision making (C) leads to frustration and loss of learning opportunities (O).^54^, ^71^ Effective and motivated learners (C) ask questions, provoke *meaning making* (M) through discussion and show commitment and initiative (C).^65^, ^124^

Supervisors use both positive and negative communication strategies (C) during *meaning making* (M) with trainees.^61^ Affirmation and respect (C) are important, particularly for more junior trainees (C),^73^, ^116^ but negative strategies (C) are more commonly reported. Rude, dismissive and aggressive communication, intimidation and harassment are features (C) of medical culture,^29^, ^74^, ^116^, ^125^, ^126^, ^127^ often directed (M) at junior members of staff by senior doctors (C).^127^ Experiencing rude dismissive communication (C) causes anger, sadness and loss of motivation (O)^29^ and can result in individuals leaving the profession (O) or engaging in harmful behaviours (O).^29^ Being bullied, scapegoated or publicly humiliated by senior doctors (C) leads to less support seeking (O) and less error disclosure (O) and therefore impacts on *safe participation in practice* (O).^80^, ^82^, ^104^

#### Feedback: mechanism and outcomes

Programme theory suggests that PME is effective when trainees receive feedback from supervisors. The extensive literature describing specific contexts and outcomes for *feedback* as a process supported its inclusion as a mechanism distinct from *meaning making* (M). In using the term *feedback* (M), we do not refer to a one‐way transmission of information from supervisor to trainee, but a mutual co‐construction of trainee performance and the means to improve it. Trainees continually receive informal comments about their work, intertwined with discussion of patient care.^95^ We focus here on these informal exchanges with supervisors, which may be part of a direct coaching process, may occur in retrospect, at morning report for example, or may relate to a longer period.^77^ Performance of procedural skills and near‐miss events may trigger *feedback* (M).^62^ Trainees may also actively seek *feedback* (M).^86^, ^124^, ^128^

*Feedback* (M) leads to *safe participation in practice* (O)^95^, ^128^ and *learning, application of theory to practice* (O).^64^, ^95^ It orients trainees to their current standard of practice,^64^, ^95^ provides direction towards areas of weakness,^129^ reinforces positive behaviours^58^ and provides a shared understanding about practice.^59^, ^86^ In the case of trainee error, *feedback* (M) is essential for future *safe participation in practice* (O).^80^, ^104^, ^122^ Lack of *feedback* (M) may be interpreted positively by trainees (O)^73^ but may also lead to less confident and competent trainees who struggle with the *application of theory to practice* (O).^130^ *Feedback* (M) from supervisors is always subject to critical review of its credibility (C) but can be very influential if it is accepted.^57^ Several papers report influential *feedback* (M) from supervisors as infrequent.^57^, ^88^, ^95^

#### Individual and interpersonal contexts for feedback: supervisor characteristics, trainee agency and trainee subjectivity

Supervisors’ characteristics and approach (C) to *feedback* (M) shape the credibility of the supervisor's comments and the receptivity of the trainee and thus the outcomes (O) of the conversation.^124^, ^128^, ^131^, ^132^ *Feedback* (M) from a respected clinician (C)^131^ with values that align with those of the trainee (C) is more likely to be accepted (O).^57^ *Feedback* (M) from a supervisor judged to lack knowledge, experience^129^ or insight into the trainee role (C)^86^ is more likely to be discounted (O). Direct observation of the trainee's work (C) enhances credibility (O).^57^, ^124^, ^129^, ^132^ Good communication skills, including clear setting of goals^90^, ^124^, ^128^, ^132^ (C), providing sufficient information to develop an action plan,^124^, ^129^, ^132^ explaining why the issue is important,^129^ allowing exchange and suggesting actions (C)^95^, ^129^ all enhance effectiveness (O). Timing^57^, ^95^, ^124^, ^129^ (C) and location^129^ (C) are also important.

Engagement and interest (agency) (C) on the part of the trainee enhance the frequency and quality of *feedback* (M) conversations.^123^, ^124^ Requesting *feedback* (M) involves a trade‐off between perceived risks and benefits. Trainees’ perceptions (subjectivity) (C) of the likely outcome of seeking *feedback* (M) influence whether they do so or not.^86^, ^128^ Those oriented more towards learning than preserving credibility (C) are more likely to actively seek *feedback* (M).^128^ Greater seniority (C) enhances comfort (C) with seeking *feedback* (M) and willingness to admit to gaps in knowledge, thereby making negative *feedback* (M) comments easier to accept (O).^86^, ^95^, ^131^ Positive (C) *feedback* (M) comments are more likely to be accepted (O) as a result of biased reasoning and a tendency to attribute negative outcomes to external factors.^86^ Openness to learning, humility and the ability to reflect (C) on negative *feedback* (M) enhances its utility (O), although an emotional response (C) to negative *feedback* (M) reduces receptivity (O).^57^, ^124^ *Feedback* (M) that aligns with trainee self‐assessment (C) is more likely to be accepted (O).^57^, ^129^

### Health systems contexts for supervisor–trainee mechanisms: work patterns, workload and work distribution

There are multiple overlapping systems’ factors that influence mechanisms of learning occurring between supervisors and trainees. Twenty‐nine papers contributed to the CMOs described in this section. Duty hour restrictions decrease the total time trainees spend at work and can increase workload for both trainees and supervisors.^17^, ^60^, ^67^, ^76^, ^133^ Fragmented work patterns (shift work),^17,71,79,96,107,123,124,130,134^ combined with short rotations,^79^, ^96^, ^123^ prevent the development of a positive supervisory relationship^79^, ^107^, ^123^, ^124^, ^135^ in which there is trust, and shared understanding and knowledge of each other's practices, values and needs.^66^, ^70^, ^72^, ^107^, ^123^, ^132^ Patient census (number of patients under a doctor's care) is related to time constraints and is frequently cited as impacting trainee learning.^17^, ^71^, ^96^, ^124^ High levels of need for patient care in terms of volume and acuity may provide rich clinical material for learning, but result in less time for elaboration and reflection.^136^ Policies mandating preceptor involvement in patient care^48^, ^71^, ^76^ and the legal responsibility of supervisors for the quality of patient care^60^, ^61^, ^67^, ^70^, ^76^ shift trainees to the periphery of practice.

*Monitoring* (M) is inhibited when supervisors do not have the opportunity to observe trainees with patients,^17^ leaving them relying on presumptive trust. This negatively impacts *entrustment* (M)***,*** reducing participation in practice and increasing faculty members’ workload.^17,60,63,70,71,79,134,135^ High patient numbers can both trigger and inhibit *entrustment*.^53^, ^60^ A positive supervisory relationship facilitates *support seeking* (M) and its absence can cause feelings of isolation (O).^72^, ^123^

Lack of relationship continuity between supervisor and trainee and heavy supervisor and trainee workloads interfere with trainee observation of seniors and thus *modelling*.^64^, ^96^, ^103^ If supervisor and trainee are not together, there is a reduction in opportunities for dialogue.^17^, ^107^, ^123^, ^130^, ^133^, ^135^ *Meaning making* is constrained,^64^, ^136^ and verbal exchange becomes restricted to that required for patient care.^17^, ^64^, ^94^, ^123^ *Feedback* is compromised as there is decreased *monitoring* (M)^87^ and limited time for conversation.^124^ Trainees may deal with multiple supervisors who do not know them over the course of a shift,^71^, ^132^ which makes it difficult to get *feedback*.^124^ Schedule asynchrony also means that trainees cannot follow patients, and supervisors who want to give *feedback* cannot do so in a timely manner.^17^, ^79^, ^130^ Trainees do not have time to reflect on *feedback* when it is received.^88^, ^95^

## Discussion

### Principal findings

We have described a realist theory of informal workplace learning between supervisors and trainees, naming three processes (supervised participation in practice, mutual observation of practice, dialogue about practice), and their six underpinning mechanisms (*entrustment, support seeking, monitoring, modelling, meaning making* and *feedback*). We have explained how contexts at individual and interpersonal levels, and local and systems levels, trigger or inhibit these mechanisms and shape their outcomes, including both key educational objectives of PME and safe patient care.

### Relationship to substantive theory

We drew on substantive theory to develop a realist theory of PME, specifically cognitive apprenticeship (CA),^39^ Billett's theory of workplace learning,^35^, ^36^, ^45^, ^137^ communities of practice (CoP)^37^, ^46^ and experience‐based learning (EBL).^38^, ^47^ All describe learners’ graduated access to practice, with peripherality being achieved by reducing risk or increasing supervision.^37^, ^46^ Additionally, CoP describes how greater participation is an outcome of demonstrating accountability (trustworthiness) through valued knowledge and behaviours. These processes are reflected in the *entrustment* mechanism. *Support seeking* does not feature significantly in the theories we considered. However, CoP does provide insight into contexts for that mechanism, describing participation as a claim to recognition as a competent community member.^37^ This sheds light on trainees’ desire for greater autonomy and their perception of threats to credibility when seeking support. The *modelling* mechanism is similar to Billett's mimetic learning (observation and imitation).^35^ Communities of practice,^37^, ^46^ EBL^47^ and Billett^35^ all identify observation of the practice of others as important in workplace learning. *Modelling*, as conceptualised in CA, involves a conscious demonstration: ‘making thinking visible’.^39^ This process is captured in the *meaning making* mechanism. *Monitoring* **,** as described in our findings, is not prominent in theory, perhaps because it is driven by patient safety more than by education outcomes.

Both CoP and EBL emphasise the importance of dialogue for learning and professional identity development.^46^, ^47^ Billett suggests that positioning individuals as constructors of knowledge is central to promoting learning through clinical practice.^35^ Our description of *meaning making* also draws on the CA concepts of *articulation* (learner verbalisation of thinking) and *reflection* (comparing performance with that of experts).^39^ Substantive theory does not distinguish between general dialogue and *feedback*; however, we have described it as a mechanism in its own right because of the substantial literature relating to it and its specific outcomes and contexts.

The outcomes we have identified are similar to those described in substantive theory, including identity development,^37^, ^38^, ^47^ demonstrable practical skills, knowledge and attitudes,^38^, ^39^, ^47^ application of theory to practice^47^ and emotional responses.^37^, ^38^, ^47^

Substantive theory is limited in the degree to which it can explain informal learning processes between supervisor and trainee in clinical environments. Cognitive apprenticeship does not account for the complexity of the workplace. Neither CoP nor Billett's work provides sufficient ‘programme specificity’, particularly in regard to contexts, to apply directly to the design and delivery of PME. Experience‐based learning shares many features with our theory; however, the contrasting roles and responsibilities of undergraduate and postgraduate learners in clinical environments are important contextual differences that limit transferability. As a middle‐range realist theory, our work fleshes out how higher‐range substantive theory applies to PME, and acknowledges the key roles of context and complexity.

### Supervision: a two‐way process

At first glance, the term *supervision* suggests a unidirectional supervisor‐led process. This emphasis on the supervisor is reflected in existing frameworks, which describe a secondary role for the trainee.^138^, ^139^ Our theory demonstrates clearly that effective PME between supervisor and trainee is a two‐way process, shaped by both, and requiring leadership from both. Substantive theory also supports this finding, emphasising both the need for support^37^, ^48^ and guidance from more experienced co‐workers^37^, ^45^ and the central role of learner agency.^35^, ^36^

Our findings echo aspects of Kilminster et al.'s^138^ and Pront et al.'s^139^ work on supervision, emphasising the importance of the relationship between supervisor and trainee, and the provision of learning opportunities within established boundaries (*entrustment*), and fostering a professional way of knowing and being (*modelling, meaning making* and *feedback*). However, supervisor competencies and behaviours are only one side of a multifaceted story. Learner subjectivity or agency can shape the outcomes of five of the six supervisor–trainee mechanisms we have described. The contribution of the learner to his or her own learning is often characterised as a matter of self‐direction, motivation and attention to the structures and requirements of training.^138^, ^140^, ^141^, ^142^ Substantive theory posits that learners’ intention and motivation can offset the weaknesses of learning environments or negate their strengths,^45^ and also determine whether learners develop an identity within a particular CoP.^37^ We have shown that learners’ interpretation of the social and cultural aspects of the clinical environment, how they ‘play the game’, shapes their learning and progress as they choose the right language, preserve credibility, adapt to variations in supervisors’ practice, and demonstrate trustworthiness and a desire to progress. This is not to diminish the impact of the supervisor on the outcomes of PME. Every mechanism we have described can be catalysed or inhibited by supervisory style and characteristics. Rather, we aim to empower trainees by highlighting that they can have a major influence on the effectiveness of their training experiences.

### Health systems contexts

Our finding that fragmented working patterns and workload can negatively influence supervisor–trainee mechanisms sheds light on the effect of restricted duty hours on PME. There continues to be little consensus on this topic, with both proponents and opponents of duty hour restrictions citing the same findings to support their positions.^34^ Reviews of the literature have shown inconsistent results, reporting no effect, negative effect and inconclusive findings.^34^, ^143^ Expert report and opinion, particularly in regard to the European Working Time Directive limit of 48 hours, is strikingly at odds with these neutral findings, identifying disruption to the supervisory relationship as a significant negative educational outcome, amongst others.^31^, ^144^, ^145^, ^146^ This divergence may be due to the metrics used to evaluate education outcomes in many of the studies published to date, which are at best proxy measures.

The complexity of PME, with its myriad contexts and mechanisms, intertwined with the complexity of patient care, makes linear cause and effect connections difficult to establish. From a theoretical perspective, legitimate peripheral participation requires access to all dimensions of practice, including building relationships with senior colleagues, which can be negatively impacted by pressure of practice.^37^, ^47^ Taking a realist approach has allowed us to focus on the process, to focus on the mechanisms that underlie PME and to describe how the operation of process is impeded when supervisors and trainees do not have adequate time to develop a supervisory relationship.

### Methodological strengths and limitations

This study was conducted in accordance with published procedural guidelines.^43^ As a middle‐range theory, the findings are sufficiently detailed and specific to PME to allow those involved in its organisation and delivery to apply them to their own context. The findings are limited by decisions made to exclude general practice, reviews and commentaries and the grey literature. Although the latter two categories have informed the study, it is possible that full inclusion would have altered or enhanced our findings. Realist synthesis involves interpretation, and each member of the team brings his or her subjectivity to bear on the process. Reflexivity was central to our approach; nonetheless, another research team might have synthesised the primary studies in a different manner. Our detailed reporting of both the methodology and findings supports the reader's understanding of how we reached our findings and judgement of their validity.

### Implications for research and practice

Our theory requires testing and refinement through empirical research. We are currently using case study methodology to explore the operation of the mechanisms in different contexts. Some of the mechanisms we have described are supported by extensive literature: *entrustment*,* modelling* and *feedback*. The others, *monitoring* and *support seeking* in particular, have been the focus of fewer studies and do not feature significantly in substantive theory. Nonetheless, high‐quality studies support their inclusion in the synthesis as complex mechanisms in themselves. We propose that these mechanisms are deserving of further research. It is the interplay among *entrustment**,** support seeking* and *monitoring*, and their contexts, rather than *entrustment* alone, that is key to trainees’ access to participation in practice and thus to the outcomes of effective PME and patient safety.

Our findings equip supervisors, trainees, hospital managers and programme directors with the knowledge needed to design, participate in and support effective PME. Both supervisors and learners need preparation for their roles in PME. The need for faculty development is well recognised, the need to prepare trainees less so. Existing short courses and guides often focus on practical tips. Although these are of value, developing an understanding of the complexity of workplace learning and providing opportunities for supervisors and trainees to reflect on how they shape learning has the potential to enhance effective PME. We have provided an accessible framework on which to build preparatory courses to allow senior doctors and trainees to conceptualise workplace learning and to maximise their own contributions.

Our findings show the importance of positive supervisory relationships, built over time, and stress the need to nurture and preserve them. With the prevailing focus on the outcomes of PME, there is a danger that the key processes that produce those outcomes and the contexts that support them will be overlooked. At a system level, we have demonstrated the negative impact of fragmented working patterns on supervisor–trainee mechanisms and their contexts. This should be a primary consideration when organising trainees’ time in the workplace. By contrast with the false dichotomy that pits service against learning in PME, we have demonstrated a clear relationship between effective PME and safe practice. This makes PME as much the mission of health systems managers as it is of programme directors.

## Contributors

DB conceived and designed the study. DB, AW and CK conducted the literature searches, screened and selected the included studies, extracted the context, mechanism and outcome configurations and undertook the synthesis. DB wrote an initial draft of the paper, to which AW and CK made substantial revisions. DB, AW and CK have all approved the final version submitted and agree to be accountable for all aspects of the work.

## Funding

this study is part of a larger project entitled Exploring Clinical Learning Environments for Postgraduate Medical Education and Training. It is funded by the Health Research Board, Ireland.

## Conflicts of interest

none.

## Ethical approval

not required.

## Supporting information


**Appendix S1.** Search strategy.

**Appendix S2.** Summary of papers included.
