# Supplementary material for: Supervised workplace learning in postgraduate training: a realist synthesis
Source: Med Educ. 2018 Aug 21;52(9):951–69. doi: 10.1111/medu.13655 (PMC6175369; doi:10.1111/medu.13655)
Supplement: Supplementary file 1 — Appendix S1. Search strategy. [file MEDU-52-951-s001.docx]

Appendix B: Summary of papers included

| **FIRST AUTHOR** | **TITLE** | **YEAR** | **PUBLICATION** | **RESEARCH AIMS** | **RESEARCH COUNTRY** | **RESEARCH DESIGN** | **SUMMARY OF FINDINGS** |
| --- | --- | --- | --- | --- | --- | --- | --- |
| Apker, Julie | Communicating professional identity in medical socialization: Considering the ideological discourse of morning report. | 2004 | Qualitative Health Research | To investigate how medical ideology and physician professional identity are socially constructed during morning report. | USA | Qualitative;  Observation;  internal medicine residents and their clinical teams | Findings indicate how, in a socialization context uniquely focused on discourse, communication functions to construct a professional identity grounded in the principles of the biomedical model. Although medical residents deviate from traditional ideology by articulating the voice of the lifeworld, faculty physicians counter these moves by asserting the voice of medicine. The authors draw conclusions regarding identity formation and the socialization practices of medical education. |
| Apramian, Tavis | Thresholds of principle and preference: Exploring procedural variation in postgraduate surgical education. | 2015 | Academic Medicine | To explore how residents make sense of, and behave in relation to, the procedural variations of faculty surgeons. | Canada | Qualitative;  Constructivist grounded theory; observation and interviews; surgical residents | The core category of the constructed theory was called thresholds of principle and preference and it captured how faculty members position some procedural variations as negotiable and others not. The term thresholding was coined to describe residents’ daily experiences of spotting, mapping, and negotiating their faculty members’ thresholds and defending their own emerging thresholds. Thresholds of principle and preference play a key role in workplace-based medical education. Postgraduate medical learners are occupied on a day-to-day level with thresholding and attempting to make sense of the procedural variations of faculty. |
| **FIRST AUTHOR** | **TITLE** | **YEAR** | **PUBLICATION** | **RESEARCH AIMS** | **RESEARCH COUNTRY** | **RESEARCH DESIGN** | **SUMMARY OF FINDINGS** |
| Apramian, Tavis | “They have to adapt to learn”: Surgeons’ perspectives on the role of procedural variation in surgical education. | 2016 | Journal of Surgical Education | To explore surgeons’’ perspectives regarding the influence of intersurgeon procedural variation on the teaching and learning of surgical residents. | Canada | Qualitative;  Grounded theory; observation and interviews;  surgeons | Surgeons endorsed the use of intersurgeon procedural variations to teach residents about adapting to the complexity of surgical practice and the norms of surgical culture. Surgeons suggested that residents’ efforts to identify thresholds of principle and preference are crucial to professional development. Principles that emerged from the study included the following: (1) knowing what comes next, (2) choosing the right plane, (3) handling tissue appropriately, (4) recognizing the abnormal, and (5) making safe progress. Surgeons suggested that learning to follow these principles while maintaining key aspects of surgical culture, like autonomy and individuality, are important social processes in surgical education. |
| Apramian, Tavis | “Staying in the game”: How procedural variation shapes competence judgments in surgical education. | 2016 | Academic Medicine | To explore how thresholds of principle and preference shaped surgeons’ intraoperative judgments of resident competence. | Canada | Qualitative; grounded theory; observation and interviews;  Surgical residents and surgeons | The core category of the study, called staying in the game, describes how surgeons make moment-to-moment judgments to allow residents to retain their role as operators. Surgeons emphasized the role of principles in making these decisions, while residents suggested that working with surgeons’ preferences also played an important role in such intraoperative assessment. |
| Balmer, Dorene | Learning behind the scenes: Perceptions and observations of role modelling in pediatric residents’ continuity experience. | 2007 | Ambulatory Pediatrics | To analyse what and how pediatric residents learn through role modelling during their continuity experience. | USA | Qualitative; observation and interviews; pediatric residents and continuity clinic preceptors | From the residents’ perspective, role modelling was an implicit and intentional learning strategy that was linked to routine clinical practice in continuity clinic. Residents learned, though modelling their CCPs, “how to talk” and “how to think things through”. Residents did not directly report modelling professional behaviour. For residents, learning through modelling was not contingent on CCPs awareness of being a role model. |
| **FIRST AUTHOR** | **TITLE** | **YEAR** | **PUBLICATION** | **RESEARCH AIMS** | **RESEARCH COUNTRY** | **RESEARCH DESIGN** | **SUMMARY OF FINDINGS** |
| Balmer, Dorene | Understanding paediatric resident-continuity preceptor relationships through the lens of apprenticeship learning. | 2008 | Medical Education | To explore the paediatric resident-continuity preceptor relationships through the lens of apprenticeship learning. | USA | Qualitative; ethnographic case study; observation and interviews, paediatric residents and primary care paediatricians | The authors observations and reports of resident learning trajectories fit well with the concept of legitimate peripheral participation. Residents learned the everyday practice of primary care as they worked alongside experienced paediatricians in the continuity clinic. Although the direction of learning was towards central participation in patient care, residents learned during transient shifts to the periphery of practice. as a function of residents’ increased participation, preceptors moved into more supportive roles. Residents were not only learners; at times, they were teachers who facilitated preceptors’ learning. |
| Balmer, Dorene | An ethnographic study of attending rounds in general paediatrics: Understanding the ritual. | 2010 | Medical Education | To investigate teaching in general paediatrics as a social phenomenon and to explore change over time in both the meaning of rounds and the context in which rounds take place. | USA | Qualitative; ethnographic case study; observation; paediatric medical teams | Four themes emerged from the data: (i) attending rounds are a pervasive and routine part of clinical education; (ii) interns, senior residents and attending physicians hold assumptions about what should happen on rounds; (iii) tension exists between interns’, senior residents’ and attending physicians’ assumptions about bedside teaching during rounds and the reality imposed by contextual factors, and (iv) bedside teaching during rounds is impacted, but not prohibited, by contextual factors. |
| Balmer, Dorene | The dance between attending physicians and senior residents as teachers and supervisors. | 2012 | Pediatrics | To examine how attending physicians and senior residents negotiated shared responsibilities for teaching and supervising on clinical work rounds. | USA | Qualitative; ethnography; observation; paediatric medical teams | Like a traditional dance with a priori choreography, and consistent with the traditional premise in graduate medical education, attending physicians frequently “stood back” and senior residents, accordingly, “stepped up” and took on teaching and supervising responsibilities. Less often, both attending physicians and senior residents assumed the lead, or attending physicians stepped up rather than entrust senior residents. The complex clinical context sometimes changed the choreography. Attending physicians and senior residents understood their mutual responsibilities but were not bound by them; they improvised to maintain high-quality patient care. |
| **FIRST AUTHOR** | **TITLE** | **YEAR** | **PUBLICATION** | **RESEARCH AIMS** | **RESEARCH COUNTRY** | **RESEARCH DESIGN** | **SUMMARY OF FINDINGS** |
| Balmer, Dorene | Learning across the explicit, implicit, and extra-curricula: An exploratory study of relative proportions of residents perceived learning in clinical areas at three paediatric residency programs. | 2015 | Academic Medicine | To investigate relative proportions of residents’ perceived learning across the explicit, implicit, and extra-curricula for six clinical learning environment review (CLER) focus areas. | USA | Qualitative; interviews; paediatric residents | Residents perceived learning to occur most often in the implicit curriculum for five of the six CLER focus areas; the one exception being health care quality, which predominantly took place in the explicit curriculum. In the implicit curriculum, role modelling and “learning by doing” were frequently reported modes of learning. The explicit curriculum was perceived as an important baseline for understanding clinical areas. Relatively less learning was perceived to occur in the extra-curriculum. |
| Bernabeo, Elizabeth | Lost in transition: The experience and impact of frequent changes in the inpatient learning environment. | 2011 | Academic medicine | To explore the experience and impact of frequent transitions on residents. | USA | Qualitative; focus groups; internal medicine residents, faculty, nurses and ancillary staff | Perceived benefits of transitions included the ability to adapts to new environments and practice styles, improved organisation and triage skills, increased comfort with stressful situations, and flexibility. Residents primarily relied on each other to cope with and prepare for transitions, with little support from the program or faculty level. Several potentially problematic workarounds were described within the context of transitions, including shortened progress notes, avoiding pages, hiding information, and sidestepping critical situations. Nearly all residents acknowledged that frequent transitions contributed to a lack of ownership and other potentially harmful effects for patient care. |
| Bhutta, Mohamed | A survey of how and why medical students and junior doctors choose a career in ENT surgery. | 2016 | The Journal of Laryngology and Otology | To ascertain determinants of an interest in a career in ENT surgery through a survey of medical students and junior doctors. | UK | Quantitative; survey; surgical foundation doctors and medical students | The most important factors that encourage ENT as a career included: the variety of operative procedures, work-life balance, inherent interest in this clinical area and inspirational senior role models. Exposure to ENT in undergraduate or postgraduate training is critical in deciding to pursue this specialty. |
| **FIRST AUTHOR** | **TITLE** | **YEAR** | **PUBLICATION** | **RESEARCH AIMS** | **RESEARCH COUNTRY** | **RESEARCH DESIGN** | **SUMMARY OF FINDINGS** |
| Bing-You, Robert | Feedback falling on deaf ears: Residents’ receptivity to feedback tempered by sender credibility. | 1997 | Medical Teacher | To characterize residents’ perceptions of effective feedback and aspects of the sender causing residents to discount such feedback. | USA | Qualitative; interviews; internal medicine residents | Well-timed, private and verbal feedback that fostered development of an action plan are examples of residents’ perceptions of effective feedback. Sender credibility, and subsequent resident receptivity to feedback, was influenced by the method of feedback delivery, the content of the feedback and the residents’ perceptions of sender characteristics, and their observation of sender behaviours. |
| Biondi, Eric | Discordance between resident and faculty perceptions of resident autonomy: Can self-determination theory help interpret differences and guide strategies for bridging the divide? | 2015 | Academic Medicine | To identify and interpret differences between resident and faculty perceptions of resident autonomy and of faculty support of resident autonomy. | USA | Mixed methods; survey and qualitative analysis of written comments; paediatric residents and faculty | The groups differed significantly on 15 of 17 parallel items but agreed that faculty sometimes provided too much direction. Written comments suggested that self-determination theory constructs were closely interrelated in residency training. Residents expressed frustration that their care plans were changed without explanation. Faculty reported reluctance to give “passive” residents autonomy in patient care unless stakes were low. Many reported granting more independence to residents who displayed motivation and competence. Some described working to overcome residents’ passivity by clarifying and reinforcing expectations. |
| Bradley, Victoria | Sticks and stones: Investigating rude, dismissive and aggressive communication between doctors. | 2015 | Clinical Medicine | To describe the extent of rude, dismissive and aggressive (RDA) communication between doctors, its context and subsequent impact. | UK | Mixed methods; survey and focus groups; junior doctors, registrars and consultants | 31% of doctors described being subject to RDA communication multiple times per week or more often, with junior and registrar doctors affected twice as often as consultants. Rudeness was more commonly experienced from specific specialties: radiology, general surgery, neurosurgery and cardiology. 40% of respondents described that RDA moderately or severely affected their working day. The context for RDA communication was described in five themes: workload, lack of support, patient safety, hierarchy and culture. Impact of RDA communication was described as personal, including emotional distress and substance abuse, and professional, including demotivation. |
| **FIRST AUTHOR** | **TITLE** | **YEAR** | **PUBLICATION** | **RESEARCH AIMS** | **RESEARCH COUNTRY** | **RESEARCH DESIGN** | **SUMMARY OF FINDINGS** |
| Chadaga, Amar | Bullying in the American graduate medical education system: A national cross-sectional survey. | 2016 | PLOS One | To deliver an estimate of bullying among residents and fellows in the United States graduate medical system and to explore its prevalence within unique subgroups. | USA | Quantitative; survey; residents and fellows | Almost half of the respondents (48%) reported being subjected to bullying although both those subjected and not subjected reported experiencing ≥ 1 bullying behaviours (95% and 39% respectively). Attendings (29%) and nurses (27%) were the most frequently identified source of bullying, followed by patients, peers, consultants and staff. Attempts to belittle and undermine work and unjustified criticism and monitoring of work were the most frequently reported bullying behaviours (44% each), followed by destructive innuendo and sarcasm (37%) and attempts to humiliate (32%). |
| Cho, Christine | Resident perspectives on professionalism lack of common consensus. | 2014 | Annals of Emergency Medicine | To characterize and understand the residents’ perspective on how professionalism develops through paediatric emergency medicine experiences. | USA | Qualitative; interviews; emergency medicine and paediatric residents | Common words associated with professionalism were “respect”, “compassion”, “empathy”, and “integrity”; however, residents did not share a common consensus. The framework for how residents described the development of their professionalism includes observations, interactions, and environment. Examples include resident observation of role models; interactions with patients, families, and co-workers; self-reflection; and the unique environment of the emergency department. Residents believed that role modelling was the most influential factor. Few reported receiving sufficient observation by attending physicians during their interactions with patients and most reported receiving little direct feedback on their professionalism. Residents descriptions of professionalism crossed multiple Accreditation Council for Graduate Medical Education (AGCME) competencies. |
| Choo, Kevin | How do supervising physicians decide to entrust residents with unsupervised tasks? A qualitative analysis. | 2014 | Journal of Hospital Medicine | To describe the factors that influence how attending and resident perceptions of trust impact decision making. | USA | Qualitative; interviews; internal medicine residents and attending physicians | The analysis yielded 535 discrete mentions of entrusting factors that were mapped to the following domains deductively, with inductively derived subthemes: trainee factors (e.g. confidence, specialty plans), supervisor factors (e.g. approachability), task factors (e.g. situational characteristics) and systems factors (e.g. workload). |
| **FIRST AUTHOR** | **TITLE** | **YEAR** | **PUBLICATION** | **RESEARCH AIMS** | **RESEARCH COUNTRY** | **RESEARCH DESIGN** | **SUMMARY OF FINDINGS** |
| Claridge, Andrew | What is the educational value of ward rounds? A learner and teacher perspective. | 2011 | Clinical Medicine | To investigate the impact on the educational value of ward rounds following the introduction of the European working time directive and foundation programme. | UK | Quantitative; survey; foundation year doctors | Eighteen percent of foundation year doctor learning occurs on ward rounds. Hindrances to learning and teaching include lack of time, increasing patient numbers and an absence of team consistency. |
| Connolly, Maureen | Variation in predictors of primary care career choice by year and stage of training. | 2003 | Journal of General Internal Medicine | To examine how role models, encouragement, and personal characteristics affect career choice at different stages (medical school vs residency) and periods (1994 vs 1997) of training. | USA | Quantitative; survey; medical students and residents | Having a primary care role model was a stronger predictor of primary care career choice for residents than for students. Likewise, peer encouragement was more predictive for residents than for students. Orientation to the emotional aspects of care was consistently associated with primary care career choice across stages and years of training. |
| **FIRST AUTHOR** | **TITLE** | **YEAR** | **PUBLICATION** | **RESEARCH AIMS** | **RESEARCH COUNTRY** | **RESEARCH DESIGN** | **SUMMARY OF FINDINGS** |
| Cope, Alexandra | “You see?” Teaching and learning how to interpret visual cues during surgery. | 2015 | Medical Education | To explore how trainees learn visual cue interpretation in the operating room. | UK | Qualitative; multiple case study; observation; surgical trainees and their trainers | Visual cue interpretation was a recurrent feature of trainer-trainee interactions and was achieved largely through the pedagogic mechanism of co-construction. Co-construction was a dialogic sequence between trainer and trainee in which they explored what they were looking at together to identify and name structures or pathology. Co-construction took two forms: ‘guided co-construction’, in which the trainer steered the trainee to see what the trainer was seeing, and ‘authentic co-construction’, in which neither trainer or trainee appeared certain of what they were seeing and pieced together the information collaboratively. Whether the co-construction activity was guided or authentic appeared to be influenced by case difficulty and trainee seniority. Co-construction was shown to occur verbally, through discussion, and also through non-verbal exchanges in which gestures made with laparoscopic instruments contributed to the co-construction discourse. |
| Cope, Alexandra | What Attitudes and Values Are Incorporated  Into Self as Part of Professional Identity  Construction When Becoming a Surgeon? | 2017 | Academic Medicine | To make explicit the attitudes and values  of a community of surgeons, with the  aim of understanding professional  identity construction within a specific  group of residents. | UK | Qualitative; grounded theory; interviews; surgeons | Participating surgeons described learning personal values or attitudes that they regarded as core to “becoming a surgeon” and key to professional identity  construction. They described learning to be a perfectionist, to be accountable, and to self-manage and be resilient. They discussed learning to be self-  critical, sometimes with the unintended consequence of seeming neurotic. They described learning effective teamwork as well as learning to take initiative and  be innovative, which enabled them to demonstrate leadership and drive actions and agendas forward within the health care organization where they worked. |
| **FIRST AUTHOR** | **TITLE** | **YEAR** | **PUBLICATION** | **RESEARCH AIMS** | **RESEARCH COUNTRY** | **RESEARCH DESIGN** | **SUMMARY OF FINDINGS** |
| Cote, Luc | How clinical teachers perceive the doctor-patient relationship and themselves as role models. | 2000 | Academic Medicine | To describe how clinicians who teach clerks and residents represent the doctor-patient relationship and how they see themselves as role models for this relationship. | Canada | Qualitative; interviews; clinical teachers | The clinical teachers identified competencies associated with the doctor-patient relationship that differed in complexity and specificity. Paramount among these competencies were the ability to conduct interviews effectively and politely, the ability to understand and involve the patient, and, in some cases, the ability to handle emotionally-charged situations. The clinical teachers tended to demand more of their students in doctor-patient relationships than they did of themselves. Lack of time and a negative attitude toward the doctor-patient relationship, on the part of both teachers and students, were obstacles to teaching and learning this essential competency, even to the point of making it difficult for teachers to demonstrate and supervise these competencies during their daily clinical activities. |
| Cote, Luc | Preceptors’ understanding and use of role modelling to develop the CanMEDS competencies in residents. | 2014 | Academic Medicine | To describe how preceptors understand and use role modelling to develop CanMEDS competencies in residents. | Canada | Qualitative; interviews; preceptors | Most participants highlighted the importance of role modelling to support residents’ development of the CanMEDS competencies, particularly communication, collaboration, and professionalism, which preceptors perceived as “less scientific” and the most difficult to teach. Although most participants reported using an implicit, unstructured role modelling process, some described more explicit strategies. Eight types of educational challenges in role modelling the CanMEDS competencies were identified, including encouraging reflective practice, understanding the competencies and their importance in one’s specialty, and being aware of one’s strengths and weaknesses as a clinical teacher. |
| **FIRST AUTHOR** | **TITLE** | **YEAR** | **PUBLICATION** | **RESEARCH AIMS** | **RESEARCH COUNTRY** | **RESEARCH DESIGN** | **SUMMARY OF FINDINGS** |
| Crebbin, Wendy | Prevalence of bullying, discrimination and sexual harassment in surgery in Australasia. | 2015 | ANZ Journal of Surgery | To describe the prevalence of inappropriate behaviours in surgical practice and training. | Australia & New Zealand | Quantitative; survey; surgical fellows, trainees, surgeons and surgical consultants | Almost half the respondents indicated that they had experienced one or more of the behaviours. This proportion was consistent across every specialty. Male surgical consultants were identified as the most likely perpetrators. More than 70% of the hospitals reported that they had instances in their organisation of discrimination, bullying or sexual harassment by a surgeon within the last 5 years. Surgical directors or surgical consultants were by far the most frequently reported perpetrators in 50% of hospitals. |
| Derrick, Sonja | The training/service continuum: Exploring the training/service balance of senior house officer activities. | 2006 | Medical Education | To explore senior house officers’ (SHOs) perception of services and training, with reference to where they place activities along the training/service continuum, and the factors that lead them to classify these activities in the way they do. | UK | Mixed methods; survey and focus groups; senior house officers | Analysis of the quantitative data allowed the construction of the training/service continuum diagram. Identified factors affecting the perceived training/service balance of SHO activities included: frequency, time, type and nature of work, number of patients, supervision, interaction, other commitments, purpose and focus of the activity, the individual trainee and trainer, and experience and competence. |
| Dijksterhuis, Marja | Assessment of competence and progressive independence in postgraduate clinical training. | 2009 | Medical Education | To explore current opinions of supervisors and trainees about how to determine when a trainee is competent to perform a clinical procedure and the role of formal assessment in this process. | Netherlands | Qualitative; focus groups; obstetrics and gynaecology trainees and supervisors | Two higher-order themes emerged: factors that determine the level of competence of a trainee in a clinical procedure, and factors that determine the level of independence granted to a trainee or acceptable to a trainee. From this study, it is evident that both determining the level of competence of a trainee for a certain professional activity and making decisions about the degree of independence entrusted to a trainee are complex, multi-factorial processes, which are not always transparent. Competence achieved in a certain clinical procedure does not automatically translate into more independent practice. |
| **FIRST AUTHOR** | **TITLE** | **YEAR** | **PUBLICATION** | **RESEARCH AIMS** | **RESEARCH COUNTRY** | **RESEARCH DESIGN** | **SUMMARY OF FINDINGS** |
| Dijksterhuis, Marja | A qualitative study on trainees’ and supervisors’ perceptions of assessment for learning in postgraduate medical education. | 2013 | Medical Teacher | To explore trainees’ and supervisors’ perceptions of what factors determine active engagement in formative assessment. | Netherlands | Qualitative; focus groups; trainees and supervisors in obstetrics and gynaecology | Three higher order themes emerged: individual perspectives on feedback, supportiveness of the learning environment and the credibility of feedback and/or feedback giver. Engaging in formative assessment with a genuine impact on learning is complex and quite a challenge to both trainees and supervisors. Individual perspectives on feedback, a supportive learning environment and credibility of feedback are all important in this process. Every one of these should be taken into account when the utility of formative assessment in postgraduate medical training is evaluated. |
| Dunkley, Lisa | Why do we choose rheumatology? Implications for future recruitment – results of the 2006 UK trainee survey. | 2008 | Rheumatology | To explore critical factors in choice of rheumatology as a specialty, and what factors might govern choices of prospective trainees. | UK | Quantitative; survey; rheumatology specialist registrars | The top four ranked factors influencing choice of rheumatology were senior house officer experience, subject matter, inspirational consultants and lifestyle aspects. Factors felt to be negatively influencing future trainees came under three key themes: poor student or postgraduate exposure, employment and service delivery issues and perceived poor profile of rheumatology. Factors positively influencing future candidates were subject matter, work/life balance, and prior exposure to the specialty. |
| Ende, Jack | Preceptors’ strategies for correcting residents in an ambulatory care medicine setting: A qualitative analysis. | 1995 | Academic Medicine | To understand the interactional strategies preceptors use as they relate to and occasionally correct interns in a general internal medicine teaching clinic. | USA | Qualitative; observation; interns and faculty preceptors in general internal medicine | The strategies the preceptors used to correct the interns were complicated and quite indirect, and tended to minimize exposing the interns’’ errors. These strategies revealed the dilemmas inherent in being a preceptor and also the beliefs the preceptors brought to their task. The preceptors’ strategies demonstrated their high regard for maintaining the inters’ self-esteem and sense of responsibility, as well as the preceptors’ willingness to forego, at least for the moment, correctional strategies that might have been more explicit and direct. |
| **FIRST AUTHOR** | **TITLE** | **YEAR** | **PUBLICATION** | **RESEARCH AIMS** | **RESEARCH COUNTRY** | **RESEARCH DESIGN** | **SUMMARY OF FINDINGS** |
| Engel, Kristen | Residents’ responses to medical error: Coping, learning, and change. | 2006 | Academic Medicine | To explore the significant emotional challenges facing resident physicians in the setting of medical mishaps, as well as their approaches to coping with these difficult experiences. | USA | Qualitative; interviews; resident physicians | Residents expressed intense emotional responses to error events. Poor patient outcomes and greater perceived personal responsibility were associated with more intense reactions and greater personal anguish. For the great majority of residents, their ability to cope with these events was dependent on a combination of reassurance and opportunities for learning. Interactions with medical colleagues and supervisory physicians were critical to this coping process. |
| Eva, Kevin | Factors influencing responsiveness to feedback: On the interplay between fear, confidence, and reasoning processes. | 2012 | Advances in Health Sciences Education | To understand the processes used by learners and physicians to interpret, accept and use data to inform their perceptions of their clinical performance, and to understand the factors believed to influence interpretation of feedback. | Multiple | Qualitative; focus groups; undergraduate learners, postgraduate learners and physicians | Multiple influences appear to impact upon the interpretation and uptake of feedback. These include confidence, experience, and fear of not appearing knowledgeable. Importantly, however, each could have a paradoxical effect of both increasing and decreasing receptivity. Less prevalent but nonetheless important themes suggested mechanisms through which cognitive reasoning processes might impede growth from formative feedback. |
| **FIRST AUTHOR** | **TITLE** | **YEAR** | **PUBLICATION** | **RESEARCH AIMS** | **RESEARCH COUNTRY** | **RESEARCH DESIGN** | **SUMMARY OF FINDINGS** |
| Farnan, Jeanne | On-call supervision and resident autonomy: From micromanager to absentee attending. | 2009 | The American Journal of Medicine | To describe clinical supervision preferences for attending physicians and residents during times of critical clinical decision-making, specifically during the on-call period; identify clinical scenarios that residents and attending physicians perceive as those requiring supervision; and provide physician in-training descriptions of the attributes of effective clinical supervisors. | USA | Mixed methods; survey and interviews; internal medicine residents and attending physicians | Findings suggest that the clinical supervision currently provided to on-call internal medicine residents is variable and highlight some strategies for improving clinical supervision during this period. Attending physicians may use ineffective and extreme strategies to supervise. For example, micromanaging attending physicians might prevent residents from fully developing their own clinical skills. |
| Friedman, Steven | Perceptions of emergency medicine residents and fellows regarding competence, adverse events and reporting to supervisors: A national survey. | 2010 | Canadian Journal of Emergency Medicine | To characterize the perceptions of emergency medicine residents and fellows of their clinical and procedural competence, as well as their attitudes, practices and perceived barriers to reporting these perceptions to their supervisors. | Canada | Quantitative; survey; emergency medicine residents and fellows | Response rates varied slightly by question; 30.5% agreed with the statement, “I sometimes feel unsafe or unqualified with undertaking unsupervised responsibilities or procedures, but I do not report this to my senior physician” and 39.5% had felt this within the past 6 months. Moreover, 41.5% reported their lack of competence to a supervisor half the time or less. Trainees reported worry about loss of trust, autonomy or respect or reputation. Nights on-call, admission decisions, and central line insertion were reported to be frequently undertaken despite not feeling competent. Suggestions to improve reporting included encouragement to report without penalty and a less judgemental environment. |
| **FIRST AUTHOR** | **TITLE** | **YEAR** | **PUBLICATION** | **RESEARCH AIMS** | **RESEARCH COUNTRY** | **RESEARCH DESIGN** | **SUMMARY OF FINDINGS** |
| Goldszmidt, Mark | Attending physician variability: A model of four supervisory styles. | 2015 | Academic Medicine | To understand variability by considering how different attendings configured and rationalised direct patient care, trainee oversights, and teaching activities. | Canada | Qualitative; grounded theory; interviews; attending physicians | Four supervisory styles were identified: direct care, empowerment, mixed practice, and minimalist. Driven by concerns for patient safety, direct care involves delegating minimal patient care responsibility to trainees. Focused on supporting trainees’ progressive independence, empowerment uses teaching and oversight strategies to ensure quality of care. In mixed practice, patient care is privileged over teaching and is adjusted on the basis of trainee competence and contextual features such as patient volume. Minimalist style involves a high degree of trust in senior residents, delegating most patient care, and teaching to them. Attendings rarely discussed the styles with the team. |
| Gonzalo, Jed | Identifying and overcoming the barriers to bedside rounds: A multicentre qualitative study. | 2014 | Academic Medicine | To identify reasons for the decrease in bedside rounds, actual barriers to bedside rounds, methods to overcome trainee apprehensions, and proposed strategies to educate faculty. | USA | Qualitative; interviews; attending physicians | Primary reasons for the perceived decline in bedside rounds were physician- and systems related, although actual barriers encountered related to systems, time, and physician-specific issues. To address resident apprehensions, six themes were identified: build partnerships, create safe learning environments, overcome with experience, make bedside rounds educationally worthwhile, respect trainee time, and highlight positive impact on patient care. Potential strategies for educating faculty were identified, most commonly faculty development initiative, divisional/departmental culture change, and one-on-one shadowing opportunities. |
| Harrison, Rebecca | Teaching internal medicine residents in the new era. | 2006 | Journal of General Internal Medicine | To provide descriptive information on the effect of resident duty-hour regulations on attendings and the educational environment. | USA | Qualitative; focus groups and survey; residents in internal medicine | Attending physicians report performing more clinical work, teaching less, using more focused teaching methods, and experiencing an increased perception of intensity. Residents observed attending physicians performing increased clinical work, being more time aware, delivering more focused teaching, and having less time to teach. Participants noted changes in autonomy and professionalism. |
| **FIRST AUTHOR** | **TITLE** | **YEAR** | **PUBLICATION** | **RESEARCH AIMS** | **RESEARCH COUNTRY** | **RESEARCH DESIGN** | **SUMMARY OF FINDINGS** |
| Hauer, Karen | How clinical supervisors develop trust in their trainees: A qualitative study. | 2015 | Medical Education | To determine how supervisors develop and experience trust in resident trainees in the clinical workplace. | USA | Qualitative; interviews; internal medicine supervisors | Supervisors characterised the meaning of trust from the perspectives of trainee competence and leadership or from their own perspective of needing to provide more or less supervision. Supervisors initially considered trust to be usually independent of prior knowledge of the resident, and then used sources of information about trust to develop their judgements of trust. Sources, which incorporated inference, included supervisors’ comparisons with a standard, direct observation of the trainee as a team leader or care provider, and stakeholder input from team members, patients and families. Barriers against and accelerators to trust formation related to the resident, supervisor, resident-supervisor relationship, context and task. Trust formation had implications for supervisors’ roles, residents’ increasingly independent provision of care, and team functioning. |
| Hoffman, Kimberley | Contextual tensions of the clinical environment and their influence on teaching and learning. | 2004 | Medical Education | To characterise how context influences clinical teaching. | USA | Qualitative; multiple case study; observation and interviews; internal medicine in-patient teams | Three tensions influenced clinical teaching: 1, patient census; 2, time sensitivity of the context; and 3, the multiple and conflicting commitments of participants. Patient census exhibited the greatest influence and was the catalyst for teaching, learning, and the allocation of total time. Time functioned as an important element influencing the pace of action, reflective and interpretative cognitive processes of the team, time available for action, and the general fatigue of the team. Conflicts among the multiple roles of ward team members disrupted individual and team teaching and learning. |
| **FIRST AUTHOR** | **TITLE** | **YEAR** | **PUBLICATION** | **RESEARCH AIMS** | **RESEARCH COUNTRY** | **RESEARCH DESIGN** | **SUMMARY OF FINDINGS** |
| Kendall, Marilyn | The learning environment for junior doctor training – what hinders, what helps. | 2005 | Medical Teacher | To explore trainees perception of trainee development during their first year of training. | UK | Qualitative; interviews; trainees | Data generated focused not only on learning outcomes, but also on important process issues. Dissatisfaction was expressed with formal and informal teaching and learning opportunities. Factors that enhance the learning environment were identified. These included being supported, a feeling of being a valued member of the team, being stretched but not over stretched, having a broad range of experiences, knowing the system, having a clear remit and being well organised. Factors inhibiting the learning environment included fractured working patterns, insufficient time with patients and seniors, as well as the converse of many of the enhancing factors. |
| Kennedy, Tara | Questioning competence: A discourse analysis of attending physicians’ use of questions to assess trainee competence. | 2007 | Academic Medicine | To explore, through discourse analysis of case presentations, the process of competence assessment for case-specific clinical independence. | Canada | Qualitative; discourse analysis; observation; emergency medicine case presentations | Questioning strategies involved clarifying questions (to ensure attendings’ understanding of the case), probing questions (to probe trainees’ understanding of a case or their underlying knowledge), and challenging questions (to challenge presuppositions). Case-related probing questions and challenging questions were found to be linguistic features of attendings’ assessment of trainee’s competence. |
| Kennedy, Tara | Clinical oversight: Conceptualizing the relationship between supervision and safety. | 2007 | Journal of General Internal Medicine | To develop a conceptual model of clinical supervision to inform and guide policy and research. | Canada | Qualitative; observation and interviews; emergency department and general internal medicine in-patient wards; physicians, residents, medical students and nurses | The term “clinical oversight” was developed to describe patient care activities performed by supervisors to ensure quality of care. “Routine oversight” (preplanned monitoring of trainees’ clinical work) can expose supervisors to concerns that trigger “responsive oversight” (a double-check or elaboration of trainees’ clinical work). Supervisors sometimes engage in “backstage oversight” (oversight of which the trainee is not directly aware). When supervisors encounter a situation that exceeds a trainee’s competence, they move beyond clinical oversight to “direct patient care”. |
| **FIRST AUTHOR** | **TITLE** | **YEAR** | **PUBLICATION** | **RESEARCH AIMS** | **RESEARCH COUNTRY** | **RESEARCH DESIGN** | **SUMMARY OF FINDINGS** |
| Kennedy, Tara | Point-of-care assessment of medical trainee competence for independent clinical work. | 2008 | Academic Medicine | To explore context-specific assessments of trainees’ competence for independent clinical work. | Canada | Qualitative; observation and interviews; internal and emergency medicine teaching team members | Supervisors’ assessment of trainee trustworthiness for independent clinical work involved consideration of four dimensions: knowledge/skill, discernment of limitations, truthfulness, and conscientiousness. Supervisors’ reliance on language cues as a source of trustworthiness data was revealed. |
| Kennedy, Tara | ‘It’s a cultural expectation…’ The pressure on medical trainees to work independently in clinical practice. | 2009 | Medical Education | To develop a theoretical exploration of the pressure on medical trainees to be independent and to generate theory-based approaches to the implications for patient safety of this pressure towards independent working. | Canada | Qualitative; grounded theory observation and interviews; teaching teams from internal and emergency medicine | Participants conceived that the pressure towards independence in clinical work originated in trainees’ desire to lay claim to the identity of a doctor (as a member of a group of autonomous high achievers), and in organisational issues such as heavy workloads and constant evaluations. |
| Kennedy, Tara | Preserving professional credibility: Grounded theory study of medical trainees’ requests for clinical support. | 2009 | British Medical Journal | To develop a conceptual framework of the influences on medical trainees’ decisions regarding requests for clinical support from a supervisor. | Canada | Qualitative; grounded theory; observation and interviews; teaching teams in internal and emergency medicine | Trainees’ decisions about whether or not to seek clinical support were influenced by three issues: the clinical question (clinical importance, scope of practice), supervisor factors (availability, approachability), and trainee factors (skill, desire for independence, evaluation). Trainees perceived that requesting frequent/inappropriate support threatened their credibility and used rhetorical strategies to preserve credibility. These strategies included building a case for the importance of requests, saving requests for opportune moments, making a plan before requesting support, and targeting requests to specific team members. |
| **FIRST AUTHOR** | **TITLE** | **YEAR** | **PUBLICATION** | **RESEARCH AIMS** | **RESEARCH COUNTRY** | **RESEARCH DESIGN** | **SUMMARY OF FINDINGS** |
| Kisiel, John | Resident physicians’ perspectives on effective outpatient teaching: A qualitative study. | 2010 | Advances in Health Sciences Education | To understand residents’ perspectives of effective outpatient teaching. | USA | Qualitative; focus groups; internal medicine residents | Leading themes were “kindness” and “teacher-learner relationships”. Junior residents were sensitive to faculty who were brusque, harsh, and degrading. Senior residents respected faculty who were humble, collaborative, and allowed residents to co-manage teaching encounters. Seniors emphasised the importance of faculty role-modelling and preferentially staffed with experts to “gain wisdom from experience”. Overall, residents expressed that effective learning requires grounded teacher-learner relationships. |
| Kroll, Leanda | Learning not to take it seriously: Junior doctors’ accounts of error. | 2008 | Medical Education | To investigate experiences of, and responses to, medical error amongst junior doctors and to examine the challenges junior doctors face and the support they receive. | UK | Qualitative; interviews; pre-registration house officers. | Errors were common and sometimes serious. In relation to disclosure and learning from error, four main themes emerged: a norm of selective disclosure; the effects of the team; individualised blame and responsibility, and the ‘learning moment’. Trainees reported disclosing errors informally, particularly when teams were seen as supportive, but were reluctant to criticise colleagues. Formal reports and disclosure to patients were very rare. Patient care was compromised when juniors did not access senior help, often when working outside their usual team environment. Lack of cooperation between teams and poor continuity of care also contributed to error. Learning was maximised when errors were formally discussed and constructive feedback offered. However, both blame and the prioritisation of reassurance over learning and structured feedback appeared to inhibit reflection on the experience of error. |
| **FIRST AUTHOR** | **TITLE** | **YEAR** | **PUBLICATION** | **RESEARCH AIMS** | **RESEARCH COUNTRY** | **RESEARCH DESIGN** | **SUMMARY OF FINDINGS** |
| LaDonna, Kori | Staging a performance: Learners’ perceptions about direct observation during residency. | 2017 | Medical Education | To explore learners’ experiences with direct observation during their residency training. | Canada | Qualitative; grounded theory; interviews; residents | Direct observation was widely endorsed as an important educational strategy, albeit one that created significant anxiety. Opaque expectations exacerbated participants’ discomfort, and participants described that being observed felt like being assessed. Consequently, participants exchanged their ‘usual’ practice for a ‘textbook’ approach; alterations to performance generated uncertainty about their role, and raised questions about whether observers saw an authentic portrayal of their knowledge and skill. |
| Larsson, Jan | Being a young and inexperienced trainee anesthetist: a phenomenological study on tough working conditions. | 2006 | Acta Anaesthesiologica Scandinavica | To investigate what difficulties trainee anaesthetists experience at work. | Sweden | Phenomenology; interviews; trainees | All trainees had experienced considerable, sometimes extreme demands at work. Most of them often felt insufficient and inadequate and had problems with the professional role. Support  from consultants was sometimes lacking. Some trainees expressed deep feelings of loneliness and helplessness in difficult  clinical situations. |
| Lingard, Lorelei | Patterns, sites of tension, and implications for novices. | 2002 | Academic Medicine | To explore the nature of communications among operating room team members from surgery, nursing, and anaesthesia to identify common communicative patterns, sites of tension, and their impact on novices. | Canada | Qualitative; observation and interviews; surgical teams | Patterns of communication were complex and socially motivated. Dominant themes were time, safety and sterility, resource, roles, and situation. Communicative tensions arose regularly in relation to these themes. Each procedure had on to four “higher-tension” events, which often had a ripple effect, spreading tension to other participants and contexts. Surgical trainees responded to tension by withdrawing from the communication or mimicking the senior staff surgeon. Both responses had negative implication for their own team relations. |
| **FIRST AUTHOR** | **TITLE** | **YEAR** | **PUBLICATION** | **RESEARCH AIMS** | **RESEARCH COUNTRY** | **RESEARCH DESIGN** | **SUMMARY OF FINDINGS** |
| Loo, Lawrence | “Page me if you need me”: The hidden curriculum of attending-resident communication. | 2012 | Journal of Graduate Medical Education | To characterize discrepancies and the types of mixed messages that are communicated to residents, as well as to assess their potential effect on resident supervision and patient safety. | USA | Quantitative; survey; internal medicine residents and attending physicians | There were clear and substantial differences between the perceptions of resident and attending physicians about when the supervising attending physician should be notified in each of the 6 vignettes. For example, 85% of attending physicians reported they wanted to be notified of an unexpected pneumothorax that required chest tube placement, but only 31% of resident physicians said they would call their attending physician during those circumstances. Common phrases such as “page me if you need me”, resulted in approximately 50% of residents reporting they would ‘rarely’ or ‘never’ call and another 41% reporting they would only ‘sometimes’ call their attending physicians. |
| Lorin, Scott | Attitudes and perceptions of internal medicine residents regarding pulmonary and critical care subspecialty training. | 2005 | Chest | To evaluate the attitudes and perceptions of internal medicine residents regarding pulmonary and critical care medicine (PCCM) training. | USA | Quantitative; survey; internal medicine residents | Key factors associated with a higher resident interest in PCCM subspecialty training included more weeks in the ICU, more role models in PCCM, and resident observations of a greater sense of satisfaction among PCCM faculty and fellows. The five most commonly cited attributes of PCCM fellowship that would attract residents to the field included intellectual stimulation (69%), opportunities to manage critically ill patients (51%), application of complex physiologic principles (45%), number of procedures performed (31%), and academically challenging rounds (29%). The five most commonly cited attributes of PCCM that would dissuade residents from the field included overly demanding responsibilities with lack of leisure time (54%), stress among faculty and fellows (45%), management responsibilities for chronically ill patients (30%), poor match of career with resident personality (24%), and treatment of pulmonary diseases (16%). |
| **FIRST AUTHOR** | **TITLE** | **YEAR** | **PUBLICATION** | **RESEARCH AIMS** | **RESEARCH COUNTRY** | **RESEARCH DESIGN** | **SUMMARY OF FINDINGS** |
| Martinez, William | Role-modelling and medical error disclosure: A national survey of trainees. | 2014 | Academic Medicine | To measure trainees’ exposure to negative and positive role-modelling for responding to medical errors and to examine the association between that exposure and trainees’ attitudes and behaviours regarding error disclosure. | USA | Quantitative; survey; residents and medical students. | The response rate was 55% (884/1,622). Training on how to respond to errors had the largest independent, positive effect on attitudes (standardized effect estimate, 0.32, P < .001); negative role-modelling had the largest independent, negative effect (standardized effect estimate, −0.26, P < .001). Positive role-modelling had a positive effect on attitudes (standardized effect estimate, 0.26, P < .001). Exposure to negative role-modelling was independently associated with an increased likelihood of trainees’ nontransparent behaviour in response to an error (OR 1.37, 95% CI 1.15–1.64; P < .001). |
| Moulton, Carol-Anne | Operating from the other side of the table: Control dynamics and the surgeon educator. | 2009 | Journal of the American College of Surgeons | To explore how academic surgeons manage and balance the often competing responsibilities of patient safety and education during the slowing-down moments. | USA | Qualitative; grounded theory; interviews; academic surgeons | An interesting control dynamic emerged as surgeons discussed the need to maintain a sense of control of an operation regardless of how much manual control they had. A dual responsibility to education and patient safety was apparent, with surgeons describing and demonstrating numerous strategies for negotiating manual control with the trainee during the critical slowing-down moments. An assessment of the trainee was implicit in the negotiation process. Numerous complications of control were identified (“bargaining”, “skidding”) as a product of this control dynamic. |
| Musselman, Laura | ‘You learn better under the gun’: Intimidation and harassment in surgical education. | 2005 | Medical Education | To compare how teachers and learners define intimidation and harassment, and to examine the impact of intimidating and harassing behaviours on the learning environment and socialisation of surgeons in training. | Canada | Qualitative; interviews; surgical faculty and residents | Interviewees acknowledged the existence of intimidation and harassment, while at the same time rationalising its occurrence. This paradox was encapsulated in participant descriptions using terms such as ‘good intimidation’. Our examination of the data helped us to understand that participants sustained the paradox of beneﬁcial intimidation and harassment by rationalising questionable behaviours on 3 speciﬁc dimensions, namely: whether an acceptable purpose could be attributed to the perpetrator; whether positive effects of the behaviour existed, and whether there was a perceived necessity for the behaviour. |
| **FIRST AUTHOR** | **TITLE** | **YEAR** | **PUBLICATION** | **RESEARCH AIMS** | **RESEARCH COUNTRY** | **RESEARCH DESIGN** | **SUMMARY OF FINDINGS** |
| Myers, Jennifer | Internal medicine and general surgery residents’ attitudes about the ACGME duty hours regulations: A multicentre study. | 2006 | Academic Medicine | To assess internal medicine and general surgery residents’ attitudes about the effects of the Accreditation Council for Graduate Medical Education duty hours regulations on medical errors, quality of patient care, and residency experiences. | USA | Quantitative; survey; internal medicine and general surgical residents | Residents reported that whereas fatigue-related errors decreased slightly, errors related to reduced continuity of care significantly increased. Additionally, duty hours regulations somewhat decreased opportunities for formal education, bedside learning, and procedures, but there was no consensus that graduates would be less well trained after duty hours reform. Residents, particularly surgical trainees, reported improvements in quality of life and reduced burnout. |
| Olmos-Vega, Francisco | Dealing with the tension: how residents seek autonomy and participation in the workplace | 2017 | Medical Education | To understanding how residents act on different affordances in the workplace is of paramount importance, as it influences their learning. | Colombia | Qualitative; focus groups and interviews; residents | Residents reported that the autonomy and practice opportunities given by their supervisors were either excessive or too limited, and both were perceived as tensions. When in excess, trainees enlisted the help of  their supervisor or peers, depending on how  safe they recognised the learning environment  to be. When practice opportunities were curtailed, trainees tried to negotiate more if they felt the learning environment was safe. When they did not, trainees became passive observers. Learning from each engagement was subject to the extent of intersubjectivity achieved between the actors involved. |
| **FIRST AUTHOR** | **TITLE** | **YEAR** | **PUBLICATION** | **RESEARCH AIMS** | **RESEARCH COUNTRY** | **RESEARCH DESIGN** | **SUMMARY OF FINDINGS** |
| Park, Jason | Observation, reflection, and reinforcement: Surgery faculty members’ and residents’ perceptions of how they learned professionalism. | 2010 | Academic Medicine | To explore perceptions of how professionalism is learned in the current academic environment. | Canada | Qualitative; interviews; surgery residents and faculty | Faculty members’ and residents’ perceptions of how they learned professionalism reflected four major themes: (1) personal values and upbringing, including premedical education experiences, (2) learning by example from professional role models, (3) the structure of the surgery residency, and (4) formal instruction on professionalism. Of these, role modelling was the dominant theme: Participants identified observation, reflection, and reinforcement as playing key roles in their learning from role models and in distinguishing the sometimes blurred boundary between positive and negative role models. |
| Pimmer, Christoph | Learning through inter- and intradisciplinary problem solving: Using cognitive apprenticeship to analyse doctor-to-doctor consultation. | 2012 | Advances in Health Sciences Education | To analyse the learning and teaching practices of interdisciplinary cooperation. | Switzerland | Qualitative; multiple case study; interviews; residents and attending physicians | The research contributes to three debates: (1) socio-cognitive and situated learning, (2) intra- and interdisciplinary learning in clinical settings, and (3), more generally, to cooperation and problem solving. Patient cases, which necessitate the cooperation of doctors in consults across boundaries of clinical specialisms, trigger intra- as well as interdisciplinary learning and offer numerous and varied opportunities for learning by requesting doctors as well as for on-call doctors, in particular those in residence. The relevance of consults for learning can also be veriﬁed from the perspective of CA which is commonly used by experts, albeit in varying forms, degrees of frequency and quality, and valued by learners. Through data analysis a model for collaborative problem-solving and help-seeking was developed which shows the interplay of pedagogical ‘methods’ of CA in informal clinical learning contexts. |
| **FIRST AUTHOR** | **TITLE** | **YEAR** | **PUBLICATION** | **RESEARCH AIMS** | **RESEARCH COUNTRY** | **RESEARCH DESIGN** | **SUMMARY OF FINDINGS** |
| Pimmer, Christoph | Contextual dynamics in clinical workplaces: Learning from doctor-doctor consultations. | 2013 | Medical Education | To explore the relationship between context and competence development in more loosely framed, day-to-day practices such as doctor-doctor consultations. | Switzerland | Qualitative; interviews; residents and attending physicians | The framework illustrates how different situational, personal and organisational factors interact in every learning situation. The interplay manifests in three different roles that doctors assume in highly dynamic ways: doctors learn as ‘actors’ (being responsible), as ‘participants’ (being involved) and as ‘students’ (being taught); contextual inﬂuences also impact on the quality of learning within these roles. |
| Ramani, Subha | “It's Just Not the Culture”: A Qualitative Study Exploring Residents' Perceptions of the Impact of  Institutional Culture on Feedback | 2017 | Teaching and Learning in Medicine | To examine residents’ perspectives on  institutional factors that affect the quality of feedback, factors that influence receptivity to feedback,  and quality and impact of faculty feedback | USA | Qualitative; focus groups; residents | Identified five key themes, dominated by resident perceptions regarding the influence of institutional feedback culture. The theme labels are taken from direct participant quotes: (a) the cultural norm lacks clear expectations and messages around feedback, (b) the prevailing culture of niceness does not facilitate honest feedback, (c) bidirectional feedback is not part of the culture, (d) faculty–resident relationships impact credibility and receptivity to feedback, and (e) there is a need to establish a culture of longitudinal professional growth. Insights: Institutional culture could play a key role in influencing the quality, credibility, and acceptability of feedback. A polite culture promotes a positive learning environment but can be a barrier to honest feedback. Feedback initiatives focusing solely on techniques of feedback giving may not enhance meaningful feedback. Further research on factors that promote feedback seeking,  receptivity to constructive feedback, and bidirectional feedback would provide valuable insights. |
| **FIRST AUTHOR** | **TITLE** | **YEAR** | **PUBLICATION** | **RESEARCH AIMS** | **RESEARCH COUNTRY** | **RESEARCH DESIGN** | **SUMMARY OF FINDINGS** |
| Reddy, Shalini | Barriers and facilitators to effective feedback: A qualitative analysis of data from multispecialty resident focus groups. | 2015 | Journal of Graduate Medical Education | To explore barriers and facilitators that residents in anaesthesiology, emergency medicine, obstetrics and gynaecology, and surgery experience with giving and receiving feedback during their clinical training/ | USA | Qualitative; focus groups | Five major themes related to feedback were  identified: teacher factors, learner factors, feedback process, feedback content, and educational context. Unapproachable attendings, time pressures due to clinical work, and discomfort with giving negative feedback were cited as major barriers in the feedback process. Learner engagement in the process was a major facilitator in the feedback process. |
| Sabri, Nessrine | The perceived effect of duty hour restrictions on learning opportunities in the intensive care unit. | 2015 | Journal of Graduate Medical Education | To evaluate residents’ perceptions of their current learning opportunities in a context of reduced duty hours, and to explore the perceived change in resident learning opportunities after call length was reduced from 24 continuous hours to 16 hours. | Canada | Quantitative; survey; residents rotating through intensive care units | Of 240 residents, 168 (70%) completed the survey. Of these residents, 63 (38%) had been exposed to both 24-hour and 16-hour call schedules. The majority of respondents (83%) reported that didactic teaching sessions held by ICU staff physicians were useful. However, of the residents trained in both approaches to overnight call, 44% reported a reduction in learner attendance at didactic teaching sessions, 48% reported a reduction in attendance at midday hospital rounds, and 40% reported a perceived reduction in self-directed reading after the implementation of the new call schedule. |
| Sheehan, Dale | Interns’ participation in learning in clinical environments in a New Zealand hospital. | 2005 | Academic Medicine | To explore factors that encourage interns to participate actively within clinical rotations. | New Zealand | Qualitative; interviews and focus groups; interns | The findings resulted in a model for participation in clinical settings where two critical components were identified: the tasks of patient care and engagement with the clinical team. These two components are further divided into two aspects: initiation and maintenance. The outcome of all four factors working well is a reinforcing cycle of activities that promote and encourage effective participation and learning. |
| **FIRST AUTHOR** | **TITLE** | **YEAR** | **PUBLICATION** | **RESEARCH AIMS** | **RESEARCH COUNTRY** | **RESEARCH DESIGN** | **SUMMARY OF FINDINGS** |
| Sheu, Leslie | How supervisors experience influences trust, supervision, and trainee learning: A qualitative study. | 2017 | Academic Medicine | To investigate how supervisor experience influences trust, supervision, and subsequently trainee learning. | USA | Qualitative; interviews; internal medicine residents and attending supervisors | Phase one: Four domains of trust and supervision varying with experience emerged: data, approach, perspective, clinical. Early supervisors were detail oriented and determined trust depending on task completion (data), were rule based (approach), drew on their experiences as trainees to guide supervision (perspective), and felt less confident clinically compared with more experienced supervisors (clinical). Experienced supervisors determined trust holistically (data), checked key aspects of patient care selectively and covertly (approach), reflected on individual experiences supervising (perspective), and felt comfortable managing clinical problems and gauging trainee abilities (clinical). Phase two: Trainees felt the exemplars reflected their experiences, described their preferences and learning needs shifting over time, and emphasized the importance of supervisor flexibility to match their learning needs. |
| Smith, Andrew | Expertise in practice: An ethnographic study exploring acquisition and use of knowledge in anaesthesia. | 2003 | British Journal of Anaesthesia | To describe and explore the way different types of knowledge are learned and used in anaesthetic practice. | UK | Qualitative; ethnography; observation and interviews; anaesthetic staff | The development of expertise in anaesthesia rests on the ability to reconcile and interpret many sources of knowledge - clinical, social, electronic, and experiential - and formal theoretical learning. Experts have mastered technical skills but are also able to understand the dynamic and uncertain condition of the anaesthetized patient and respond to changes in it. This expertise is acquired by working with colleagues, and, importantly, by working independently, to develop personal routines. Routines mark the successful incorporation of new knowledge but also function as a defence against the inherent uncertainty of anaesthetic practice. The habits seen in experts' routines are preferred ways of working chosen from a larger repertoire of techniques which can also be mobilized as changing circumstances demand. |
| **FIRST AUTHOR** | **TITLE** | **YEAR** | **PUBLICATION** | **RESEARCH AIMS** | **RESEARCH COUNTRY** | **RESEARCH DESIGN** | **SUMMARY OF FINDINGS** |
| Stegeman, Jantine | Knowing and acting in the clinical workplace: Trainees’ perspectives on modelling and feedback. | 2013 | Advances in Health Sciences Education | To explore modelling and feedback in two disparate clinical disciplines, surgery and paediatrics. | Netherlands | Qualitative; interviews; surgery and paediatric trainees | Our conclusion on modelling is: modelling is a dynamic and fragmented process reﬂecting discipline bound characteristics and working styles. On feedback it is: ‘feedback’ serves as vehicle for three distinctive forms of commenting on performance, each holding a speciﬁc power of expression for learning. We propose to view clinical workplace learning as: an interactive master-apprenticeship model encompassing modelling and feedback as natural educational routes. We conceptualise modelling and feedback as ‘function’ of interaction (developing grounded theory). Modelling function and feedback function may serve to study these routes as didactical components of ongoing interaction between trainer and trainee rather than an educator-driven series of unrelated events. |
| Sterkenburg, Anneke | When do supervising physicians decide to entrust residents with unsupervised tasks? | 2010 | Academic Medicine | To investigate factors guiding clinical supervisors’ decisions to trust residents with critical patient-care tasks. | Netherlands | Mixed methods; survey and interviews; anaesthetic residents and attending anaesthetists | Thirty-two attending anaesthetists and 31 residents answered the questionnaire (response rate 58%), and 10 participants from each group were interviewed. Attendings varied in their opinions regarding how much independence to give residents, particularly postgraduate year (PGY) 2, 3, and 4 residents. PGY1 residents reported working above their expected level of competence but estimate their own ability as sufficient, whereas PGY5 residents reported working below their expected level of competence. The authors classified factors that determine entrustment into four groups: characteristics of the resident, the attending, the clinical context, and the critical task. |
| **FIRST AUTHOR** | **TITLE** | **YEAR** | **PUBLICATION** | **RESEARCH AIMS** | **RESEARCH COUNTRY** | **RESEARCH DESIGN** | **SUMMARY OF FINDINGS** |
| Sternzus, Robert | Resident role modelling: “It just happens”. | 2015 | Academic Medicine | To understand residents’ perceptions of themselves as role models, describe how residents learn about role modelling, and identify ways to improve resident role modelling. | Canada | Qualitative; interviews; residents in internal medicine, general surgery, and paediatrics | Four primary themes were identified through data analysis: residents perceived role modelling as the demonstration of “good” behaviours in the clinical context; residents believed that learning from their role modelling “just happens” as long as learners are “watching”; residents did not equate role modelling with being a role model; and residents learned about role modelling from watching their positive and negative role models. |
| Stewart, Jane | To call or not to call: A judgement of risk by pre-registration house officers. | 2008 | Medical Education | To explore what influences a junior doctors’ response to a judgement call within a clinical setting and to describe the relationships between these influences. | UK | Qualitative; grounded theory; interviews; pre-registration house officers | The data demonstrated a number of inﬂuences on whether junior doctors chose to seek senior assistance. These included the upholding and balancing of tenets that were necessary for ensuring safe practice, and estimating the chance and severity of potential negative consequences to patients, themselves and their teams. In order to make these judgements, junior doctors drew on different forms of knowledge, especially knowledge gained from previous clinical experiences. In judging whether or not to contact a senior, pre-registration house ofﬁcers (PRHOs) were practising essential clinical attributes, that of independent yet co-operative and discerning practitioners who are able to balance multiple considerations while ensuring patient care. |
| Tallentire, Victoria | Understanding the behaviour of newly qualified doctors in acute care contexts. | 2011 | Medical Education | To investigate the factors that influence the behaviour of junior doctors and to develop a framework that promotes understanding of this important area. | UK | Qualitative; grounded theory; focus groups; specialist registrar, foundation year 1 and 2 doctors | Six main themes, grouped under three broad headings, emerged from the data: ‘transferring knowledge into practice’ and ‘decision making and uncertainty’ (cognitive challenges); ‘acts and omissions’ and ‘identity and expectations’ (roles and responsibilities), and, ﬁnally, ‘the medical hierarchy’ and ‘performing under stress’ (environmental factors). The framework presented within this paper illustrates the complex relationships between these factors. |
| **FIRST AUTHOR** | **TITLE** | **YEAR** | **PUBLICATION** | **RESEARCH AIMS** | **RESEARCH COUNTRY** | **RESEARCH DESIGN** | **SUMMARY OF FINDINGS** |
| Taylor, Christine | The influence of mentorship and role modelling on developing physician-leaders: Views of aspiring and established physician-leaders. | 2009 | Journal of General Internal Medicine | To understand the role and functions of mentoring and role-modelling in developing physician-leaders as experienced by aspiring and established physician-leaders. | USA | Qualitative; interviews; faculty | Twenty-five Cleveland Clinic faculty participated (14 established physician–leaders, 11 aspiring leaders). Three themes emerged: 1. Role modelling was differentiated as a valued experience separate from mentoring, with respondents describing the significant influence of purely observational learning and “watching leaders-in-action”. 2. Many respondents favoured a series of “strategic” interactions with various individuals about specific professional issues rather than traditional, longitudinal mentoring experiences. 3. Emotional and psychological support was considered the most valued type of interventional activity. |
| Teman. Nicholas | Entrustment of general surgery residents in the operating room: Factors contributing to provision of resident autonomy. | 2014 | Journal of the American College of Surgeons | To determine the factors contributing to faculty decisions to grant residents autonomy in the operating room, the barriers to granting this autonomy, and the factors that facilitate entrustment. | USA | Quantitative; survey; attending surgeons | There were 116 attending surgeons who responded to the survey (49%). Factors most important to increasing resident responsibility and autonomy in the operating room were the residents observed clinical skill and the attending surgeon’s confidence level with the operation. Factors believed to prevent awarding graduated responsibility and autonomy in the operating room included an increased focus on patient outcomes, a desire to increase efficiency and finish operations earlier, and expectations of attending surgeon involvement by the hospital and patients.  Among themes discerned in faculty responses to an open-ended question about the greatest  challenges in graduate surgical education, 47% of faculty identified work-hour regulations/  time restrictions. Fourteen percent pointed to a change to a shift-work mentality and decreased  ownership of responsibility for patients by residents; 13% described a lack of resident autonomy due to increased supervision requirements. |
| **FIRST AUTHOR** | **TITLE** | **YEAR** | **PUBLICATION** | **RESEARCH AIMS** | **RESEARCH COUNTRY** | **RESEARCH DESIGN** | **SUMMARY OF FINDINGS** |
| Ten Cate, Olle | Entrustment decision making in clinical training. | 2016 | Academic Medicine | To explore the entrustment decision-making process in health care training. | Netherlands | Qualitative; focus groups; | The authors discuss theoretical backgrounds and terminology of trust and entrustment in the clinical workplace. The competency-based movement and the introduction of entrustable professional activities force educators to rethink the grounds for assessment in the workplace. Anticipating a decision to grant autonomy at a designated level of supervision appears to align better with health care practice than do most current assessment practices. The authors distinguish different modes of trust and entrustment decisions and elaborate five categories, each with related factors, that determine when decisions to trust trainees are made: the trainee, supervisor, situation, task, and the relationship between trainee and supervisor. The authors’ aim in this article is to lay a theoretical foundation for a new approach to workplace training and assessment. |
| Teunissen, Pim | Attending doctors’ perspectives on how residents learn. | 2007 | Medical Education | To develop a theoretical framework of learning in the clinical workplace by adding the perspective of attending doctors. | Netherlands | Qualitative; grounded theory; interviews; attending doctors in obstetrics and gynaecology | Three related themes emerged. The ﬁrst concerned the central role of participation in work-related activities: according to attending doctors, residents learn by tackling the everyday challenges of clinical work. The second involved the ways in which attending doctors inﬂuence what residents learn from work-related activities. The ﬁnal theme focused on attending doctors’ views of the essential characteristics of residents and their development during residency. |
| Teunissen, Pim | How residents learn: Qualitative evidence for the pivotal role of clinical activities. | 2007 | Medical Education | To seek insight into the intricate process of how residents learn in the clinical workplace. | Netherlands | Qualitative; grounded theory; focus groups; obstetrics and gynaecology residents | An underlying theoretical framework emerged from the data, which clariﬁed what happens when residents learn by doing in the clinical workplace. This framework shows that work-related activities are the starting point for learning. The subsequent processes of ‘interpretation’ and ‘construction of meaning’ lead to reﬁnement and expansion of residents’ knowledge and skills. Interaction plays an important role in the learning process. |
| **FIRST AUTHOR** | **TITLE** | **YEAR** | **PUBLICATION** | **RESEARCH AIMS** | **RESEARCH COUNTRY** | **RESEARCH DESIGN** | **SUMMARY OF FINDINGS** |
| Teunissen, Pim | Who wants feedback? An investigation of the variables influencing residents’ feedback-seeking behaviour in relation to night shifts. | 2009 | Academic Medicine | To investigate what individual and situational variables influence residents’ feedback-seeking behaviour on night shifts. | Netherlands | Quantitative; survey; obstetrics and gynaecology residents | The response rate was 76.5%. Results showed that residents who perceive more feedback benefits report a higher frequency of feedback inquiry and monitoring. More perceived feedback costs result mainly in more feedback monitoring. Residents with a higher learning goal orientation perceive more feedback benefits and fewer costs. Residents with a higher performance goal orientation perceive more feedback costs. Supportive physicians lead residents to perceive more feedback benefits and fewer costs. |
| Tilburt, Jon | How do doctors use information in real-time? A qualitative study of internal medicine resident precepting. | 2007 | Journal of Evaluation in Clinical Practice | To describe information exchange behaviour by internal medicine residents and attendings in ambulatory resident clinic precepting rooms. | USA | Qualitative; observation; internal medicine residents and attendings | Four themes of information exchange behaviour emerged: (i) questioning behaviours that were used as part of the communication process in which the resident and attending doctor could reason together; (ii) searching behaviour of non-human knowledge sources occurred in a minority of precepting interactions; (iii) unsolicited knowledge offering and (iv) answering behaviours were important means of exchanging information. |
| **FIRST AUTHOR** | **TITLE** | **YEAR** | **PUBLICATION** | **RESEARCH AIMS** | **RESEARCH COUNTRY** | **RESEARCH DESIGN** | **SUMMARY OF FINDINGS** |
| Tiyyagura, Gunjan | The greater good: How supervising physicians make entrustment decision in the paediatric emergency department. | 2014 | Academic Pediatrics | To understand how supervisors determine the level of procedural supervision to provide a resident, taking into consideration simulation performance; to understand factors that affect supervisors’ transparency to parents about residents’ procedural experience. | USA | Qualitative; interviews; supervisors in paediatrics | Five factors influenced supervisors’ entrustment decisions: 1) resident characteristics that include self-reported confidence, seniority, and prior interactions with the resident; 2) supervisor style; 3) nature of the procedure/characteristics of the patient; 4) environmental factors; and 5) parental preferences. Supervisors thought that task-based simulators provided practice opportunities but that simulated performance did not provide evidence for entrustment. Supervisors reported selectively omitting details about a resident’s experience level to families to optimize experiential learning for residents they entrusted to perform a procedure. |
| Tsouroufli, Maria | Consultant medical trainers, modernising medical careers (MMC) and the European time directive (EWTD): Tensions and challenges in a changing medical education context. | 2008 | BMC Medical Education | To explore consultant trainers’ views on postgraduate medical education and the implications of cultural changes, resulting from MMC and EWTD, aiming to identify impediments in the successful implementation of MMC, within a context of reduced working hours. | UK | Qualitative; interviews; hospital consultants | Consultant Trainers felt that new working patterns resulting from the EWTD and MMC have changed the nature of medical education. Loss of continuity of care, reduced clinical exposure of medical trainees and loss of the popular apprenticeship model were seen as detrimental for the quality of medical training and patient care. Consultant Trainers' perceptions of medical education were embedded in a traditional medical education culture, which expected long hours' availability, personal sacrifices and learning without formal educational support and supervision. Over-reliance on apprenticeship in combination with lack of organisational support for Consultant Trainers' new  responsibilities, resulting from the introduction of MMC, and lack of interest in pursuing training in  teaching, supervision and assessment represent potentially significant barriers to progress. |
| **FIRST AUTHOR** | **TITLE** | **YEAR** | **PUBLICATION** | **RESEARCH AIMS** | **RESEARCH COUNTRY** | **RESEARCH DESIGN** | **SUMMARY OF FINDINGS** |
| Wallenburg, Iris | Learning to doctor: Tinkering with visibility in residency training. | 2013 | Sociology of Health and Illness | To explore the coexistence of multiple practices of residents’ visibility in daily clinical routines. | Netherlands | Qualitative; ethnography; obstetrics and gynaecology wards | The article lists four visibilities: staging residents, negotiating supervision, playing the invisibility game and ﬁlming surgical operations. The article shows how attending physicians and medical residents tinker with these visibilities in daily clinical work to provide good care while enacting learning space, highlighting the increasing importance of visualising technologies in clinical work. Moreover, the article contributes to traditional sociological accounts on medical education, shifting the focus from medical education as a social institution to the practices of medical training itself. Such a focus on practice helps to gain an understanding of how the current reform challenges clinicians’ educational activities. |
| Watling, Christopher | Learning from clinical work: The roles of learning cues and credibility judgements. | 2012 | Medical Education | To explore experiences considered by doctors to be influential in their learning in order to better understand this process. | Canada | Qualitative; grounded theory; interviews; academic doctors | A model of clinical learning emerged in which the clinical work itself is central. As they observe and participate in clinical work, learners can attend to a variety of sources of information that facilitate the interpretation of the experience and the construction of knowledge from it. These ‘learning cues’ include feedback, role models, clinical outcomes, patient or family responses, and comparisons with peers. The integration of a cue depends on the learner’s judgement of its credibility. Certain cues, such as clinical outcomes or feedback from patients, are seen as innately credible, whereas other cues, particularly feedback from supervisors, are subjected to critical judgement. |
| Watling, Christopher | ‘Sometimes the work just needs to be done’: Socio-cultural influences on direct observation in medical training. | 2016 | Medical Education | To explore the influence of professional culture on the use of direct observation within medical education. | Canada | Qualitative; grounded theory; interviews; residents | Observation was used selectively; specialties tended to observe the clinical acts that they valued most. Two cultural values consistently challenged the direct observation across specialties: (i) autonomy in learning and (ii) efﬁciency in health care provision. Direct observation was a primarily learner driven activity, which left learners caught in the middle, wanting observation but also wanting to appear independent and efﬁcient. |
| **FIRST AUTHOR** | **TITLE** | **YEAR** | **PUBLICATION** | **RESEARCH AIMS** | **RESEARCH COUNTRY** | **RESEARCH DESIGN** | **SUMMARY OF FINDINGS** |
| Wear, Delese | Hidden in plain sight: The formal, informal, and hidden curricula of a psychiatry clerkship. | 2009 | Academic Medicine | To examine perceptions of the formal, informal, and hidden curricula in psychiatry as they are observed and experienced by (1) attending physicians who have teaching responsibilities for residents and medical students, (2) residents who are taught by those same physicians and who have teaching responsibilities for medical students, and (3) medical students who are taught by attendings and residents during their psychiatry rotation. | USA | Qualitative; focus groups; attendings, residents and students | All three groups offered a similar belief that the knowledge, skills, and values of the formal curriculum focused on building relationships. Similarly, all three suggested that elements of the informal and hidden curricula were expressed primarily as the values arising from attendings’ role modelling, as the nature and amount of time attendings spend with patients, and as attendings’ advice arising from experience and intuition versus “textbook learning.” Whereas students and residents offered negative values arising from the informal and hidden curricula, attendings did not, offering instead the more positive values they intended to encourage through the informal and hidden curricula. |
| Weissmann Peter | Role modelling humanistic behaviour: Learning bedside manner from the experts. | 2006 | Academic Medicine | To study how excellent clinical teachers impart the behaviours and attitudes consistent with humanistic care to their learners. | USA | Qualitative; observation; clinical faculty | Clinical teachers taught primarily by role modelling. Although they were highly aware of their significance as role models, they did not typically address the human dimensions of care overtly. Despite the common themes of role modelling identified, each clinical teacher exhibited unique teaching strategies. These clinical teachers identified self-reflection as the primary method by which they developed and refined their teaching strategies. |
| **FIRST AUTHOR** | **TITLE** | **YEAR** | **PUBLICATION** | **RESEARCH AIMS** | **RESEARCH COUNTRY** | **RESEARCH DESIGN** | **SUMMARY OF FINDINGS** |
| Wright, Scott | Examining what residents look for in their role models. | 1996 | Academic Medicine | To determine which characteristics were deemed most important by residents regarding their physician role models. | USA | Quantitative; survey; residents | A total of 195 residents (85%) responded. Most of the residents (74%) were satisfied with the proportions of positive role models in their current residency training programmes. Clinical skills, personality, and teaching ability were rated the three most important factors in selecting a staff physician as a role model. When the residents were asked to recall the positive role models encountered while in medical school, attending physicians in internal medicine received the highest scores. |
| Wright, Scott | Attributes of excellent attending-physician role models. | 1998 | The New England Journal of Medicine | To identify attributes that distinguish excellent role models from their colleagues. | USA | Quantitative; survey; physicians | Of the 341 attending physicians who responded, 144 (42 percent) had been identified as excellent role models. Having greater assigned teaching responsibilities was strongly associated with being identified as an excellent role model. In the multivariate analysis, five attributes were independently associated with being named as an excellent role model: spending more than 25 percent of one’s time teaching (odds ratio, 5.12; 95 percent confidence interval, 1.81 to 14.47), spending 25 or more hours per week teaching and conducting rounds when serving as an attending physician (odds ratio, 2.48; 95 percent confidence interval, 1.15 to 5.37), stressing the importance of the doctor–patient relationship in one’s teaching (odds ratio, 2.58; 95 percent confidence interval, 1.03 to 6.43), teaching the psychosocial aspects of medicine (odds ratio, 2.31; 95 percent confidence interval, 1.23 to 4.35), and having served as a chief resident (odds ratio, 2.07; 95 percent confidence interval, 1.07 to 3.98). |
| **FIRST AUTHOR** | **TITLE** | **YEAR** | **PUBLICATION** | **RESEARCH AIMS** | **RESEARCH COUNTRY** | **RESEARCH DESIGN** | **SUMMARY OF FINDINGS** |
| Wright, Scott | Which values do attending physicians try to pass on to house officers? | 2001 | Medical Education | To determine the values and attitudes which attending physicians try to pass on to residents in order to encourage their professional development. | USA | Quantitative; survey; attending physicians | Of the 341 attending physicians who returned a completed questionnaire, 265 (78%) shared the single value or attitude they try to pass on to residents. The  four main categories into which more than 95% of responses could be categorized were: (i) caring, (ii) respect, (iii) communication and (iv) integrity. There were no statistically significant differences between the responses given by attending physicians who had been named as excellent role models and their colleagues who had not been so named. |
| Wright, Scott | Excellence in role modelling: Insight and perspectives from the pros. | 2002 | Canadian Medical Association Journal | To better understand role modelling by examining the insights of respected physician role models. | USA | Qualitative; interviews; internal medicine attending physicians | The informants identified specific characteristics related to role modelling. Subcategories under the domain of personal qualities included interpersonal skills, a positive outlook, a commitment to excellence and growth, integrity and leadership. Under the domain of teaching, the subcategories were establishing rapport with learners, developing specific teaching philosophies and methods, and being committed to the growth of learners. Subjects thought there was some overlap between teaching and role modelling, but felt that the latter was more implicit and more encompassing. Being a strong clinician was regarded as necessary but not sufficient for being an exemplary physician role model. Perceived barriers to effective role modelling included being impatient and overly opinionated, being quiet, being overextended, and having difficulty remembering names and faces. Physician role models described role modelling consciousness, in that they specifically think about being role models when interacting with learners. Subjects believed that medical learners should emulate multiple role models. |
| **FIRST AUTHOR** | **TITLE** | **YEAR** | **PUBLICATION** | **RESEARCH AIMS** | **RESEARCH COUNTRY** | **RESEARCH DESIGN** | **SUMMARY OF FINDINGS** |
| Wyber, Rosemary | For better or worse: Role models for New Zealand house officers. | 2007 | The New Zealand Medical Journal | To examine the positive and negative role modelling experiences of New Zealand house officers. | New Zealand | Qualitative; interviews; house officers | The interviews revealed three broad relationships that house officers consider important for identifying their role models: the relationship between house officer and the model; the model’s relationship with patients; and the model’s relationship with medicine. Clinical skills are excluded from this discussion because they are generally a poor demarcation between positive and negative role models. |
| Yasigi, Alexandre | Clinical teachers as role models: Perceptions of interns and residents in a Lebanese medical school. | 2006 | Medical Education | To identify the characteristics and learning impact of role models as perceived by interns and residents. | Lebanon | Quantitative; survey; interns and residents | A total of 88 responders (97%) had positive role models and 87 responders (96%) had negative role models in their current training programme. Characteristics identiﬁed most frequently and ranked most highly by the trainees were related to clinical skills in positive role models and to inadequate humanistic and collaborative attitudes in negative models. Role modelling had a positive impact on the achievement of clinical skills for 55% of the responders, and on the acquisition of humanistic and collaborative attitudes for 30% of them. Thirty-eight per cent of the trainees were inﬂuenced by their role models in the choice of their specialities. Responses were generally comparable between levels of training and between medical and surgical specialities. |
