# Supplementary material for: Supervised workplace learning in postgraduate training: a realist synthesis
Source: Med Educ. 2018 Aug 21;52(9):951–69. doi: 10.1111/medu.13655 (PMC6175369; doi:10.1111/medu.13655)
Supplement: Supplementary file 2 — Appendix S2. Summary of papers included. [file MEDU-52-951-s002.docx]

# **Appendix A: Search Strategy**

**General Search Steps**:

1. Graduate *AB
2. Postgraduate *AB
3. Medical education
4. Medical training
5. Residency *AB
6. Internship *AB
7. 1 OR 2 (Postgraduate OR graduate)
8. 3 OR 4 (Medical education OR medical training)
9. 5 OR 6 (Residency OR internship)
10. 7 AND 8
11. 8 AND 9
12. 10 OR 11
13. Clinical
14. Learning
15. Environment
16. Work place
17. Workplace
18. 13 AND 14 (Clinical AND Learning)
19. 15 AND 18
20. 16 OR 17
21. 14 AND 20
22. 19 OR 21
23. 12 AND 22

**Specific search steps**: **EWTD**

1. Graduate *AB
2. Postgraduate *AB
3. Medical education
4. Medical training
5. Residency *AB
6. Internship *AB
7. 1 OR 2 (Postgraduate OR graduate)
8. 3 OR 4 (Medical education OR medical training)
9. 5 OR 6 (Residency OR internship)
10. 7 AND 8
11. 8 AND 9
12. 10 OR 11
13. European working time directive
14. EWTD
15. Duty hours
16. 13 OR 14
17. 15 OR 16
18. 12 AND 17

**Specific search steps: Role modelling**

1. Graduate *AB
2. Postgraduate *AB
3. Medical education
4. Medical training
5. Residency *AB
6. Internship *AB
7. 1 OR 2 (Postgraduate OR graduate)
8. 3 OR 4 (Medical education OR medical training)
9. 5 OR 6 (Residency OR internship)
10. 7 AND 8
11. 8 AND 9
12. 10 OR 11

13) Role model*

14) 12 AND 13

**Specific search steps: Career Choice**

1. Graduate *AB
2. Postgraduate *AB
3. Medical education
4. Medical training
5. Residency *AB
6. Internship *AB
7. 1 OR 2 (Postgraduate OR graduate)
8. 3 OR 4 (Medical education OR medical training)
9. 5 OR 6 (Residency OR internship)
10. 7 AND 8
11. 8 AND 9
12. 10 OR 11
13. Career
14. Choice
15. 13 AND 14
16. 12 AND 15

**Specific search steps: Supervision**

1. Graduate *AB
2. Postgraduate *AB
3. Medical education
4. Medical training
5. Residency *AB
6. Internship *AB
7. 1 OR 2 (Postgraduate OR graduate)
8. 3 OR 4 (Medical education OR medical training)
9. 5 OR 6 (Residency OR internship)
10. 7 AND 8
11. 8 AND 9
12. 10 OR 11
13. AB Supervis*
14. 12 AND 13
